# Duckweed: a starch-hyperaccumulating plant under cultivation with a combination of nutrient limitation and elevated CO_2_

**DOI:** 10.3389/fpls.2025.1531849

**Published:** 2025-02-10

**Authors:** Ling Guo, Yang Fang, Songhu Wang, Yao Xiao, Yanqiang Ding, Yanling Jin, Xueping Tian, Anping Du, Zhihua Liao, Kaize He, Shuang Chen, Yonggui Zhao, Li Tan, Zhuolin Yi, Yuqing Che, Lanchai Chen, Jinmeng Li, Leyi Zhao, Peng Zhang, Zhengbiao Gu, Fangyuan Zhang, Yan Hong, Qing Zhang, Hai Zhao

**Affiliations:** ^1^ CAS Key Laboratory of Environmental and Applied Microbiology, Environmental Microbiology Key Laboratory of Sichuan Province, National Engineering and Research Center for Natural Medicines, Chengdu Institute of Biology, Chinese Academy of Sciences, Chengdu, China; ^2^ Department of Pediatrics, Children Hematological Oncology and Birth Defects Laboratory, The Affiliated Hospital of Southwest Medical University, Sichuan Clinical Research Center for Birth Defects, Southwest Medical University, Luzhou, China; ^3^ Analytical and Testing Center, Sichuan University of Science and Engineering, Zigong, China; ^4^ Key Laboratory of Eco-environments in Three Gorges Reservoir Region (Ministry of Education), SWU-TAAHC Medicinal Plant Joint R&D Centre, School of Life Sciences, Southwest University, Chongqing, China; ^5^ School of Ecology and Environmental Sciences & Yunnan Key Laboratory for Plateau Mountain Ecology and Restoration of Degraded Environments, Yunnan University, Kunming, China; ^6^ Department of Biology, Pitzer College, Claremont, CA, United States; ^7^ National Key Laboratory of Plant Molecular Genetics, CAS Center for Excellence in Molecular Plant Sciences, Chinese Academy of Sciences, Shanghai, China; ^8^ School of Food Science & Technology, Jiangnan University, Wuxi, China; ^9^ College of Food and Bioengineering, Xihua University, Chengdu, China

**Keywords:** duckweed, high-efficiency starch producer, artificial cultivation, “source” to “sink”, weak “flow”

## Abstract

**Introduction:**

The increasing global demand for starch has created an urgent need to identify more efficient and sustainable production methods. However, traditional starch sources, such as crop-based options, experience significant bottlenecks due to limitations in land use, water consumption, and the impacts of climate change. Therefore, there is a pressing need to explore and develop new sources of starch.

**Methods:**

We develop a novel duckweed cultivation technology that combines nutrients limitation and CO_2_ supplementation to achieve very high starch content. In this study, we integrated whole-genome sequencing, epigenomics, transcriptomics, enzyme activity, and composition variation to elucidate the mechanisms of efficient starch accumulation in duckweed in terms of starch accumulation and carbon partitioning, regulation of the expression of genes in the starch metabolic pathway, and sucrose biosynthesis and transportation.

**Results and discussion:**

Although *Landoltia punctata* exhibits dramatic gene family contraction, its starch content and productivity reached 72.2% (dry basis) and 10.4 g m^-2^ d^-1^, respectively, in 10 days, equivalent to a yield of 38.0 t ha^-1^ y^-1^, under nutrient limitation treatment with elevated CO_2_ levels. We also examined the mechanism of high starch accumulation in duckweed. This phenomenon is associated with the regulation of DNA methylation and transcription factors as well as the significantly upregulated transcription levels and the increased activities of key enzymes involved in starch biosynthesis. Moreover, while nitrogen redistribution was increased, sucrose biosynthesis and transportation and lignocellulose biosynthesis were reduced. These alterations led to a reduction in lignocellulose and protein contents and ultimately an increase in the accumulation of starch in the chloroplasts.

**Conclusion:**

This work demonstrates the potential of duckweed as a highly efficient starch producer.

## 1 Introduction

Starch plays a pivotal role in human society. It provides 80% of the world’s calories (Bahaji et al., 2014; Keeling and Myers, 2010; Liu et al., 2018) while serving as a raw material for the biochemical industry and for biofuel production (Smith, 2008). Starch primarily originates from the starch-storing organs of staple crops (Keeling and Myers, 2010), which accounts for 60–70% of starch storage (Rao and Annadana, 2017). Thus, starch productivity is highly correlated with crop yield (Bahaji et al., 2014).The estimated annual yield of staple crops is 2.5 billion tons worldwide (Zeeman et al., 2010), and these crops contain three main types of starch-storing organs: cereal grains (e.g., corn, wheat, rice, and barley), roots and tubers (e.g., potato, sweet potato, yam, and cassava), and beans (Zeeman et al., 2010). However, these organs only constitute a portion of the whole crop plant (Zhu et al., 2010), whereas the other parts, such as the stems and leaves, are agricultural residues and wastes that may become environmental pollutants. Cereal grains, as the most important crop component, are the main sources of starch. The development of cereal grains, as seed organs, depends on sexual reproductive growth. This process, which includes flowering, pollination, and grain filling, can be easily disrupted by biotic and abiotic stresses (Dolferus et al., 2011). Therefore, stable production of cereals and beans has been a challenge. Furthermore, although the starch biosynthetic pathway has been well studied, genetic manipulations to significantly increase starch productivity remain difficult due to the complexities of starch metabolic networks (Bahaji et al., 2014; Zeeman et al., 2010). With the continuous growth of the world’s population, the demand for staple crops is predicted to rise by 70–100% by 2050 (Foley et al., 2011; Godfray et al., 2010). A sustainable staple crop supply has been and always will be a challenge. Therefore, new approaches for highly efficient starch production with new starch crops are urgently needed.

Duckweed, a floating aquatic monocot, is one of the fastest-growing higher plants on earth (Ziegler et al., 2015). Lacking stems, it consists of “frond” structures and few or no roots. Biomass accumulates through asexual budding and vegetative growth processes (Fu et al., 2017; Maheshwari and Chauhan, 1963; Pieterse, 2013). Its biomass can increase nearly exponentially, and its estimated yield reaches 55 t ha^-1^ per year (dry weight, DW) (Oron, 1994). Duckweed is a feed source for domestic animals, fishes, and even indigenous people in Southeast Asia (Bhanthumnavin and Mcgarry, 1971). Duckweed has attracted extensive attention because of its potential application in feed/food, bioenergy production, and wastewater treatment (Fourounjian et al., 2020). Research has shown that the starch content of duckweed can reach 75% on a sugar substrate (Reid and Bieleski, 1970). In particular, under culture conditions without organic carbon, the starch content can reach 48% after 10 days of treatment (sugar-free solution) (Liu et al., 2015b). These findings indicate its potential as a new starch crop for biofuel conversion and food supplementation. It is necessary to further improve the starch production capacity, elucidate the mechanism of starch accumulation, and evaluate the potential of duckweed.

Herein, we develop a novel duckweed cultivation technology that requires limited nutrients and CO_2_ supplementation to achieve very high starch content (72.2%) and extremely efficient production. In this study, we integrated whole-genome sequencing, epigenomics, transcriptomics, enzyme activity, and composition variation. With these methods, we elucidated the mechanisms of efficient starch accumulation in duckweed in terms of starch accumulation and carbon partitioning, regulation of the expression of genes in the starch metabolic pathway, and sucrose biosynthesis and transportation.

## 2 Experimental section

### 2.1 Plant material

*Landoltia punctata* strain 0202 was originally obtained from Xinjin, China (N 30°24′46.74″, E 103°48′34.08″) and stored at the Chengdu Institute of Biology, Chinese Academy of Sciences (Chengdu, China). The stored duckweed was precultured in 1/5 Hoagland medium (Hoagland and Arnon, 1937) in containers (23×14×4.5 cm^3^) for 7−10 days under a 16 h/8 h (light/dark) photoperiod at 25°C/15°C with a light intensity of 110 μmol photons m^-2^ s^-1^ in a greenhouse.

### 2.2 Cultivation of duckweed with nutrient limitations and/or elevated CO_2_ levels

The cultivation of duckweed was conducted in 500 ml beakers (90 mm outer diameter ×120 mm height) containing 500 mL medium (1/5 Hoagland medium or deionized water) with an initial inoculation of 1 g fresh precultivated duckweed. This experiment included three treatment conditions: nutrient limitation (L), cultivation of duckweed in deionized water; elevated CO_2_ level (C), with a CO_2_ supply of 2500 ± 100 ppm (**Supplementary Table S1**) (Chen et al., 2017; Zhu et al., 2008); and the combination of L and C (LC). With the three variables listed above, all the duckweed was cultivated for 10 days under a 24 h/0 h (light/dark) photoperiod at 25°C with a light intensity of 110 μmol photons m^-2^ s^-1^. Fresh duckweed (0.5 g) from each sample was collected at 0 h, 2 h, 5 h, 9 h, 24 h, 48 h, 72 h, 120 h, 168 h, and 240 h and snap-frozen immediately in liquid nitrogen. The samples were stored at -80°C for subsequent biophysiological and biochemical analysis, transcriptome sequencing, and/or whole-genome bisulfite sequencing (WGBS). Three biological replicates were performed to acquire the mean values for all data in the experiment, with the exception of WGBS.

On a pilot scale, duckweed was treated under LC conditions for one month (from 14 February to 13 March 2014) beside Dianchi Lake, southwestern Kunming, E 102°47′, N 24°51′). Approximately 4.0 kg of fresh duckweed was transferred into 3.1×4.5×0.4 m^3^ (W×L×D) tanks filled with tap water in a greenhouse where CO_2_ was aerated to a concentration of 2500 ± 100 ppm. Duckweed was cultivated at 20−30°C with sunlight during the day and a fluorescent lamp at night for 4 days (**Supplementary Table S2**).

### 2.3 Light microscopy and transmission electron microscopy

Duckweed fronds in the treatment and control groups were fixed, embedded, and dehydrated as described previously (Wu and Messing, 2010). Semithin sections were stained with 0.2% (w/v) KI/I_2_ solution and observed under a Motic BA210 microscope equipped with a digital camera (**Supplementary Figure S1**). Ultrathin sections (80 nm thick) of duckweed were cut with an ultramicrotome (Leica EM UC7, Leica) and observed under a transmission electron microscope (Hitachi H-7650TEM, Japan) (Cao et al., 2016). The images were processed (sharpened, brightened, and contrast adjusted) and assembled using Photoshop CS6 (Adobe).

### 2.4 Gene family analysis

The 8 species from which we collected protein sets to conduct gene family analysis are as follows: *Klebsormidium flaccidum (* Hori et al., 2014 *)*, *Zostera marina (* Olsen et al., 2016 *)*, *Arabidopsis thaliana (* Arabidopsis Genome, 2000 *)*, *Oryza sativa* japonica (Goff et al., 2002), *Zea mays (* Schnable et al., 2009 *)*, *Spirodela polyrhiza (* Wang et al., 2014 *)*, *Landoltia punctata*, and *Lemna minor (* Van Hoeck et al., 2015 *)*. OrthoMCL (Li et al., 2003) (mcl –I 1.5) was used to delineate gene families using the results from the ‘all-versus-all’ BLASTP (e-value threshold 1 × 10^-3^) comparison.

### 2.5 Transcriptome analysis

Total RNA was extracted using an OMEGA™ Plant DNA/RNA Kit (OMEGA, USA) following the manufacturer’s instructions. Genomic DNA was removed by DNase I (Fermentas, USA). The RNA concentration, quality, and integrity number (RIN) were measured with an Agilent 2100 Bioanalyzer (Agilent, USA). Pair-end sequencing (2×150 bp) using the Illumina HiSeq 2500 platform at Mega Genomics Co. (Beijing, China) was conducted on the libraries.

All raw sequences were evaluated using FastQC_v0.11.3 (http://www.bioinformatics.babraham.ac.uk/projects/fastqc/), where low-quality sequences (reads with adapters, ambiguous ‘N’ bases, or low-quality scores) were filtered out. The high-quality clean reads were aligned to the ribosomal RNA (rRNA) database using Bowtie2 (Langmead and Salzberg, 2012) to remove rRNA reads. Then, HISAT2-2.0.5 (Jang et al., 2015) was used to align the reads to the reference genome, and Stringtie-1.3 (Pertea et al., 2015) was used to calculate the FPKM values. Significant differences in expression levels were evaluated using Ballgown_2.6.0 (Pertea et al., 2016) (p value ≤ 0.05, |log_2_(fold change)| ≥ 0.58).

Twenty differentially expressed genes (DEGs) were validated by quantitative reverse transcription−PCR (qRT−PCR) analysis. Total RNA was extracted from backup samples for transcriptome analysis using the Eastep^®^ Super Total RNA Extraction Kit (Promega, USA). Reverse transcription was performed using the GoScript™ Reverse Transcription System (Promega, USA). qRT−PCR was performed using UltraSYBR Mixture (CWBiotech, China) with a CFX Connect Real-Time PCR System (Bio-Rad). *Actin* was used as the reference gene. The primers used are listed in **Supplementary Table S4**. Three qRT−PCR technical replicates were conducted for each sample.

### 2.6 Quantification of the expression of key genes

The expression of key genes involved in CO_2_ fixation, carbon concentration, and starch synthesis (*PEPC*, *Rubisco*, *UGPase*, *AGPase*, *SSS*, and *GBSS*) was quantified via qRT−PCR. qRT−PCR was performed according to the protocol described above, with *Actin* as the reference gene, and three technical replicates were performed. The primers used are listed in **Supplementary Table S5**.

### 2.7 Subcellular localizations of enzymes and translocators

To explore the subcellular localization of the target proteins, the coding regions of the corresponding genes in *Landoltia punctata* were first independently cloned and inserted into the binary vector pCAMBIA2300-GFP, which was then independently transformed into *Agrobacterium* strain GV3101. Suspension cells of *Lemna gibba* were infiltrated with *Agrobacterium* strain GV3101, which carried either the GFP-fused C-terminus or N-terminus of the target protein or an empty vector (control) (Koroleva et al., 2005). After infiltrating the suspension of cells with *Agrobacterium* for 3 days, protoplasts were generated via digestion with Cellulase R10 and Macerozyme R10 (Yakult Pharmaceutical Ind. Co., Ltd., Japan) (Yoo et al., 2007). All of the fluorescence signals were detected using a confocal laser scanning microscope (Leica TCS SP8). The excitation/emission spectra were 488/493 to 598 for GFP and 633/647 to 721 for chlorophyll autofluorescence.

### 2.8 DNA methylation analysis

Following the manufacturer’s instructions for the Acegen Bisulfite-Seq Library Prep Kit (Acegen, Shenzhen, China Cat. #BS0311-48), a WGBS library was constructed using 500 ng of purified genomic DNA spiked with 0.1% (w/w) unmethylated Lambda DNA (Promega, Madison, WI). Briefly, the DNA was sonicated (Covaris) to a mean fragment size distribution of 200–400 bp. The fragmented DNA was end-repaired, 5’-phosphorylated, 3’-dA-tailed, and ligated to adapters. The adapter-ligated DNA molecules were purified using 1× Agencourt AMPure XP magnetic beads and subjected to bisulfite conversion using the ZYMO EZ DNA Methylation-Gold Kit (Zymo, Cat. #D5005). Libraries were then amplified by PCR using 20 μL of bisulfite-converted DNA molecules, 25 μL of KAPA HiFi HotStart Uracil+ ReadyMix, and 5 μL of 8-bp index primers, each with a final concentration of 1 μM. PCR was performed under cycle conditions of initial denaturation at 98°C for 1 min; 10 cycles of 98°C for 15 s, 60°C for 30 s, and 72°C for 30 s; and extension for 1 min at 72°C. The constructed WGBS libraries were then analyzed using an Agilent 2100 Bioanalyzer and quantified using a Qubit fluorometer with a Quant-iT dsDNA HS Assay Kit (Invitrogen).

Pair-end sequencing (2×150 bp) was performed on WGBS libraries using the Illumina HiSeq X Ten platform at Mega Genomics Co. (Beijing, China). All the raw sequences were evaluated by FastQC_v0.11.3 (http://www.bioinformatics.babraham.ac.uk/projects/fastqc/), where low-quality sequences (reads with adapters, 5% ambiguous bases ‘N’, or low-quality scores) were filtered out. Clean reads were aligned against the *Landoltia punctata* genome using the BSMAP 2.90 (Xi and Li, 2009) with the default parameters. The identification of methylated cytosine positions for each sample was performed independently in accordance with a previous study (Lister et al., 2009). The CG, CHG, and CHH methylation rates of genes were determined using AWK script.

The samples subjected to methylation inhibitor treatment were sent to Basebio Co. (Chengdu, China) for WGBS. WGBS library construction, quality control, and sequencing (Illumina HiSeq 4000) were performed in accordance with the methods described above. Clean reads were aligned against the reference genome using WALT (H. Chen et al., 2016) with the default parameters and then deduplicated before downstream analysis. MethPipe (Song et al., 2013) was used to identify sites of methylation where at least five reads containing cytosine were considered. A binomial test was performed for each cytosine base to check the methylated cytosine (mC) site, with a false discovery rate of ≤ 0.05. The methylation level (ML) of each target region was calculated with Eq. (1) using ViewBS (Huang et al., 2018) as follows:


(1) $$\text{ML}=\frac{reads(mC)}{reads(mC)+reads(C)}$$

### 2.9 Analytical methods

#### 2.9.1 Composition analysis

Prior to analysis, the duckweed was dried to a constant weight at 60°C and milled. Structural carbohydrates, including glucan, xylan, galactan, arabinan, mannan, lignin, and ash, were determined according to the methods recommended by the National Renewable Energy Laboratory, USA (Sluiter et al., 2012). The starch content was determined via hydrolysis of duckweed with HCl, as described previously (Liu et al., 2015b). The cellulose content was calculated by subtracting starch from glucan. Xylan, galactan, arabinan, and mannan together are considered hemicellulose. Lipid was extracted using diethyl ether with a Soxtec system with reference to AOAC 920.39 B (http://down.foodmate.net/standard/sort/10/25070.html). Total Kjeldahl nitrogen (TKN) was measured using a FOSS KJ2200 System (FOSS Corp., Denmark). The protein content was calculated as the TKN content multiplied by the conventional factor (6.25). Pectin was extracted according to the methods described by (Luo et al., 2016) and (Yang et al., 2011) and determined according to the methods described by (Blumenkrantz and Asboe-Hansen, 1973), with GalUA (Sigma−Aldrich) used as a standard. The contents of carbon and nitrogen were determined using an elemental analyser (Vario EL Cube; Elementar Analysensysteme GmbH, Germany).

#### 2.9.2 Enzyme activity assay

Fresh duckweed (0.5 g) was homogenized in 5 ml of precooled enzyme extraction solution (100 mM tricine-NaOH (pH 8.0), 8 mM MgCl_2_, 2 mM EDTA, 50 mM 2-mercaptoethanol, 12.5% (v/v) glycerol, and 5% (w/v) insoluble polyvinylpyrrolidone-40) (Nakamura et al., 1989). The homogenate was subsequently centrifuged at 13,400 × *g* for 10 min at 4°C. The resulting supernatant was subsequently used to measure the enzyme activities. The activities of AGPase (EC 2.7.7.27) and starch synthase (SSS, EC 2.4.1.21; GBSS, EC 2.4.1.242) were analyzed using the methods described by Nakamura et al. (1989). The activities of the two enzymes were tested by measuring the change in NADH at 340 nm using a microplate reader (Thermo Scientific Varioskan Flash, Thermo Fisher Scientific Inc., USA). The activities of α-amylase (EC 3.2.1.1) and β-amylase (EC 3.2.1.2) were estimated following previously described methods (Liu et al., 2015b). Rubisco (EC 4.1.1.39) activity was tested as described by Sharkey et al. (1991) using a spectrophotometric diagnostic kit (Suzhou Comin Biotechnology Co., Ltd., Suzhou, China). PEPC (EC 4.1.1.31), NADP-MDH (EC 1.1.1.82), and malic enzyme (ME, EC 1.1.1.39) activities were assayed according to Gonzalez et al. (1984) and Johnson and Hatch (1970) using a spectrophotometric diagnostic kit (Suzhou Comin Biotechnology Co., Ltd., Suzhou, China). Enzyme activity definitions: AGPase and SS activity: One unit is defined as the production of 1 nmol NADPH per mg protein per minute. Amylase activity: One unit is defined as the hydrolysis of starch to produce 1 mg of maltose per minute. Rubisco activity: One unit is defined as the oxidation of 1 nmol NADH per mg protein per minute at 25°C. PEPC activity: One unit is defined as the consumption of 1 nmol NADH per mg protein per minute. NADP-MDH activity: One unit is defined as the consumption of 1 nmol NADPH per mg protein per minute. NADP-ME activity: One unit is defined as the production of 1 nmol NADPH per mg protein per minute.

#### 2.9.3 Determination of intracellular sucrose

Sucrose was extracted according to the method described by (Zhu et al., 2018). The duckweed was dried to a constant weight at 60°C and then powdered using a pulverizer. Then, 50 mg of duckweed powder was suspended in 1 ml of deionized water and sonicated for 30 min. The mixture was incubated at 80°C for 1 h with intermittent shaking every 5 min. The sample was then centrifuged at 13,400 × *g* for 20 min at 4°C. The supernatant was stored at -20°C. The residue was resuspended in 1 ml of deionized water for a second round of extraction. The supernatants from two rounds of extraction were pooled and filtered through a 0.45-μm-pore size filter.

The sucrose content was determined using a high-performance liquid chromatography (HPLC) system (Thermo 2795, Thermo Corp.) equipped with an evaporative light scattering detector (ELSD) (All-Tech ELSD6000, All-tech., Corp.). The samples were separated on an Aminex HPX-87P column (300 × 7.8 mm) at 79°C using ultrapure water as the mobile phase at 0.6 ml min^-1^. Analytical pure sucrose (AR) was used as a standard.

#### 2.9.4 Determination of Glycerate 3-P

The 3-PGA content was measured via an enzymatic assay described by Flores-Tornero et al. (2017). First, 3-PGA was extracted using precooled methanol/chloroform (1:1, v/v). Then, the endogenous enzymes in the samples were heat-inactivated at 70°C for 10 min. Next, 20 μL of the resulting extract was added to 980 μL of reagent (0.1 M Tris-HCl (pH 7.6), 5 mM MgCl_2_, 40 μM NADH, 2 mM ATP, 6 units of PGK and 3 units of GAPDH). The mixture was incubated at 25°C for 20 min. Finally, the samples were measured at 340 nm using a spectrophotometer. In the control, 20 μL of methanol/chloroform (1:1, v/v) instead of the extract was added to the mixture.

### 2.10 Identification of transcription factors involved in the starch biosynthetic pathway in *Landoltia punctata* by gene coexpression analysis

The transcriptome data associated with nutrient limitation and elevated CO_2_ levels (2500 ± 100 ppm) (LC0, LC1, and LC3) were used for coexpression analysis (Zeng et al., 2018). The transcription factor library of *Landoltia punctata* was constructed with iTAK (Zheng et al., 2016). The absolute value of the Pearson correlation coefficient between the key genes of the starch biosynthetic pathway (*AGPase, SSS, GBSS*, and *GBE*) and TFs was calculated using the Hmisc package (Harrell, 2018). The top 30 genes for which the absolute value of the Pearson correlation coefficient was greater than 0.8 are listed in **Supplementary Data Sheets S1**-**S17** with the FPKM values of the TFs.

## 3 Results and discussion

### 3.1 A simple technology for efficiently producing starch in duckweed

**Figure 1:**
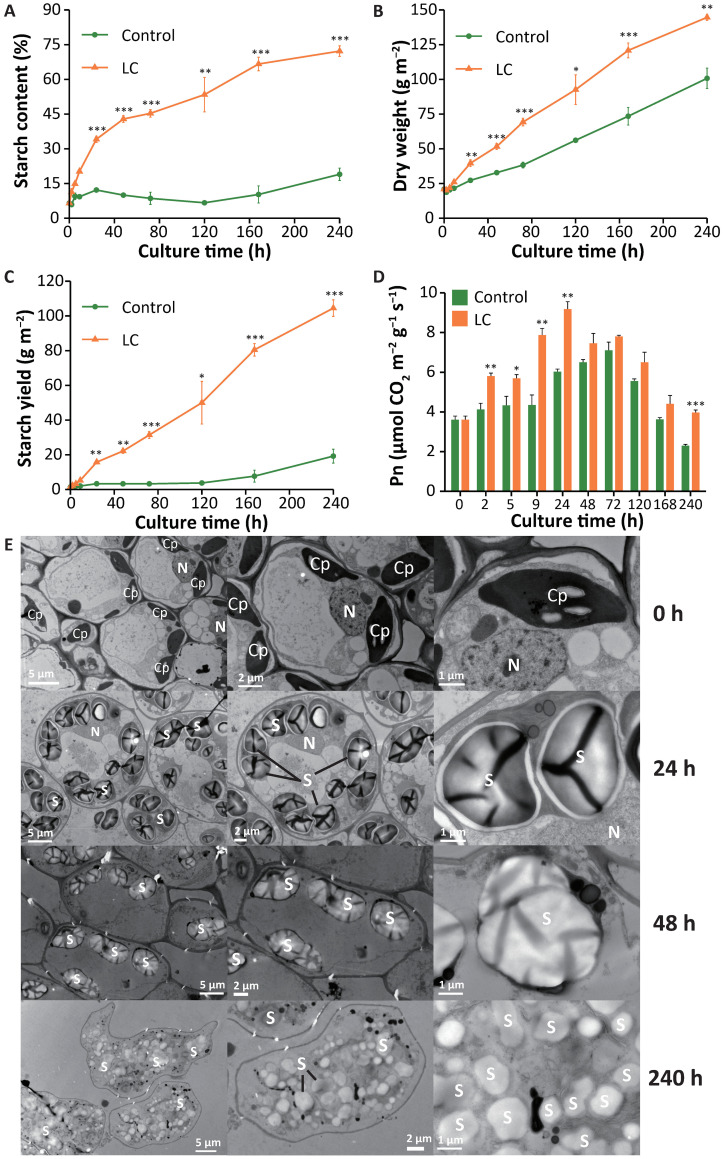
Accumulation of starch in *Landoltia punctata* under the LC treatment. (**A-D),** Changes in **(A)** starch content, **(B)** dry weight, **(C)** starch yield and **(D)** net photosynthetic rate during 240 hours of cultivation. The control was cultivated in 1/5 Hoagland medium. LC, cultivated under conditions of nutrient limitation and elevated CO_2_ (2500 ± 100 ppm). Pn, net photosynthetic rate. The error bars represent the standard deviations measured from three independent cultures. The asterisks indicate statistically significant differences between the data from each treatment group and those from the control group under the same assay conditions (Student’s *t* test). *P<0.05; **P<0.01; ***P<0.001. **(E)** Starch granules in duckweed fronds observed by transmission electron microscopy (TEM). Duckweed was cultivated under the LC treatment. The fronds were sampled at 0, 24, 48 and 240 h and then fixed, embedded, and dehydrated prior to observation via TEM. Cp, chloroplast; S, starch; N, nucleus.

We developed a simple technology that makes duckweed an efficient starch producer. *Landoltia punctata 0202* was previously identified as a useful duckweed ecotype with high potential for starch accumulation (Liu et al., 2015a, Liu et al., 2015b; Tao et al., 2013). We used limited nutrients (“L” conditions by cultivating duckweed in deionized water) and an elevated concentration of CO_2_ (“C” conditions by supplying CO_2_ to 2500 ± 100 ppm) to stimulate biomass accumulation and starch production (Appl. No. ZL201710855019.8) (Guo et al., 2020). This technology greatly improves the starch content, enhances biomass accumulation, and dramatically increases the starch yield in duckweed. The starch content increased from 7 ± 0% to 72 ± 2% (dry basis, d.b.) (**Figure 1A**). The biomass of duckweed reached 145 ± 2 g m^-2^ (DW) in 10 days, whereas that of the control was only 101 ± 7 g m^-2^ (**Figure 1B**). A net amount of 104 g of starch was produced per square meter (**Figure 1C**), equivalent to 38.0 t ha^-1^ y^-1^, which is greater than that of almost all storage organs of crop plants (Jacques et al., 2003; Ray et al., 2013). There have been no reports that nutrient limitation can simultaneously increase the starch content and yield of cereals. Studies on model plants have also indicated that although nutrient limitation can increase starch content, it markedly reduces plant biomass at the same time (Hermans et al., 2006).

**Figure 2:**
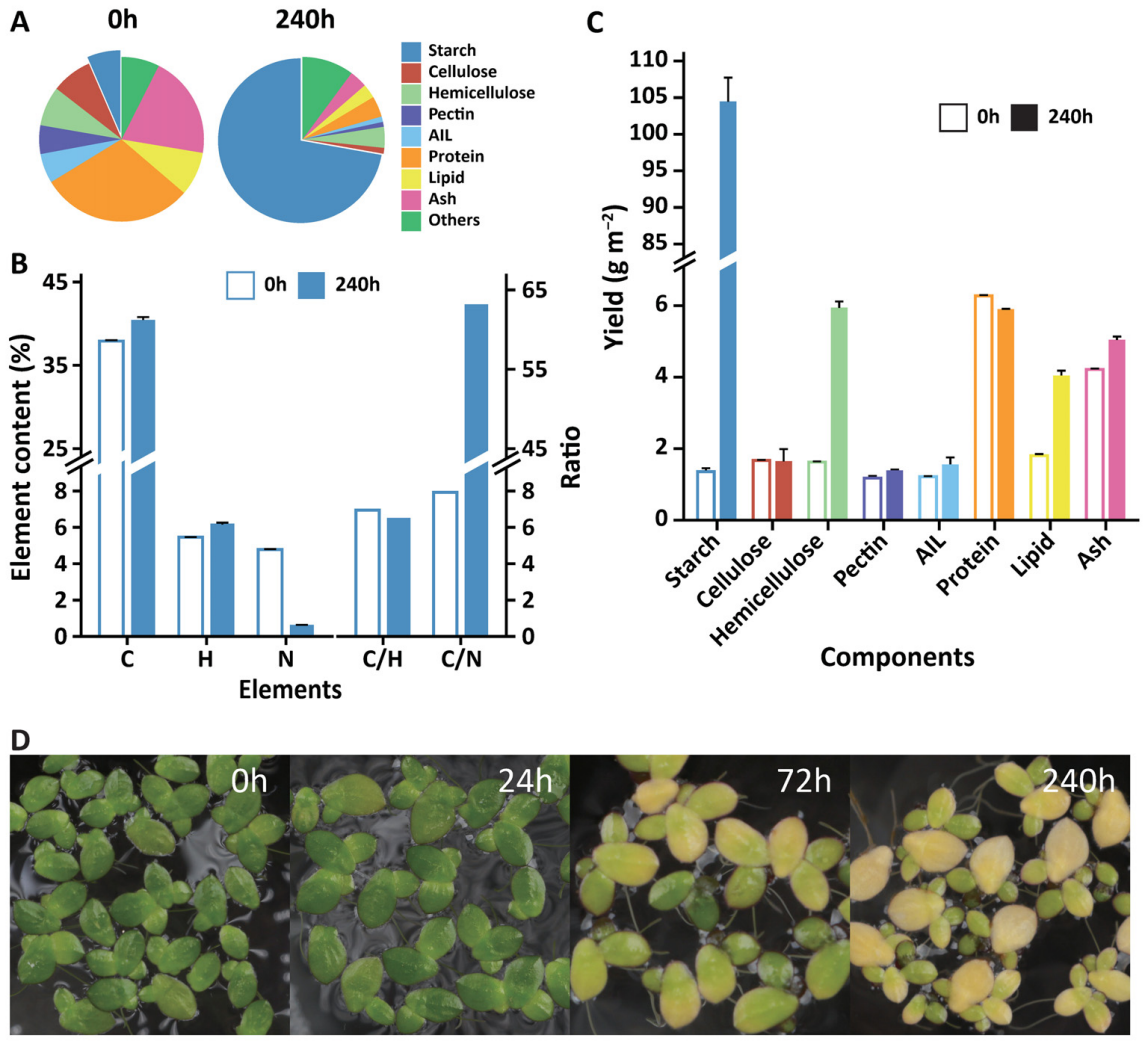
Changes in the content and yield of the primary composition in *Landoltia punctata* under the LC treatment. **(A)** Changes in the contents of primary compounds in duckweed before treatment and 240 hours after treatment. AIL, acid insoluble lignin. Lipid was extracted using diethyl ether. **(B)** Changes in primary element contents and their ratios in duckweed before treatment and 240 hours after treatment. C, carbon; H, hydrogen; N, nitrogen; C/H, ratio of carbon to hydrogen; C/N, ratio of carbon to nitrogen. **(C)** Changes in the yields of the primary compounds in duckweed before treatment and 240 hours after treatment. The error bars represent the standard deviations measured from three independent cultures. **(D)** Fresh fronds at different culture times under treatment.

During the cultivation process, the fronds of duckweed gradually became larger and distinctively yellow under limited nutrient and CO_2_ supplementation (LC) treatment (**Figure 2D**). The moisture content gradually decreased from 91 ± 1% at the beginning to 69 ± 1% at the 10th day (**Supplementary Figure S2**).

Moreover, the net photosynthetic rate (Pn) of duckweed initially increased but then gradually decreased, and the Pn of the treatment group was always greater than that of the control group within the 10-day period (**Figure 1D**) (Leakey et al., 2009).

### 3.2 Starch accumulation and carbon partitioning

#### 3.2.1 Expression and activities of key enzymes in the starch biosynthetic pathway

**Figure 3:**
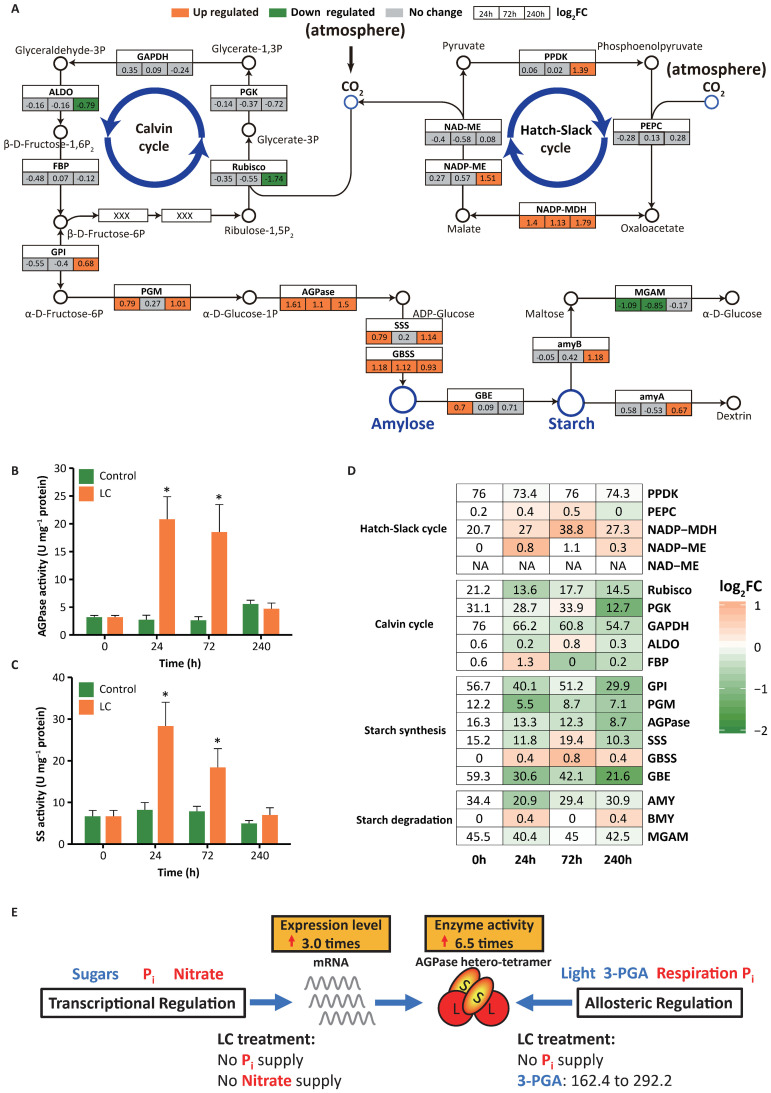
Expression and DNA methylation of genes involved in starch metabolism in *Landoltia punctata* under the LC treatment. **(A)** Expression of key genes involved in CO_2_ fixation, carbon concentration, starch biosynthesis, and starch degradation. Boxes colored in orange or cyan indicate the genes whose expression was upregulated or downregulated, respectively (p value ≤ 0.05, |Log_2_(fold change)| ≥ 0.58), after cultivation for 24 h, 72 h, and 240 h compared with that at 0 h. The numbers in the box represent the log_2_FC values. **(B, C)** Changes in the activities of key enzymes in the starch biosynthesis pathway at different culture times under treatment. AGPase, ADP−glucose pyrophosphorylase; SS, starch synthase, including both soluble starch synthase (SSS) and granule-bound starch synthase (GBSS). The error bars represent the standard deviations measured from three independent cultures. Asterisks indicate significant differences compared with the control (evaluated by one-way analysis of variance (ANOVA)). The unlabeled data are not significant. *P<0.05. **(D)** DNA methylation rates in the 2 kb upstream region of key genes in the CG context involved in CO_2_ fixation, carbon concentration, starch biosynthesis, and starch degradation. The numbers in the boxes indicate the methylation rates (%). The color gradient indicates the log_2_FC of the methylation rate compared with that at 0 h, where FC is the fold change. **(E)** Schematic diagram of AGPase activity regulation in duckweed under treatment. AGPase activity is regulated at the transcriptional and allosteric levels through multiple environmental factors (3-PGA, Pi, and nitrate) under LC treatment. The blue font and arrows indicate activation, the red font indicates inhibition, and the red arrows indicate increased expression or enzyme activity. 3-PGA, μg g^-1^ FW.

The transcript levels of all the key starch biosynthesis genes in the chloroplast were upregulated under the LC treatment (**Figure 3A**). Plastidial ADP−glucose pyrophosphorylase (AGPase) (**Supplementary Figure S6**), the first rate-limiting enzyme in starch biosynthesis, determines carbon flux into starch to a large extent. AGPase is precisely regulated at the transcriptional and posttranslational levels, including allosteric regulation and redox modulation (Geigenberger, 2011; Streb and Zeeman, 2012). Previous studies revealed that AGPase activity is induced by increased 3-phosphoglycerate (3-PGA) and decreased phosphate through allosteric regulation (Sokolov et al., 1998; Tiessen et al., 2002) and suppressed by phosphate (Nielsen et al., 1998) and nitrate (Scheible et al., 1997) through transcriptional regulation. In our study, elevated CO_2_ significantly increased the 3-PGA content from 162.4 μg g^-1^ fresh weight (FW) to 292.2 μg g^-1^ FW (**Supplementary Figure S10A**). Nutrient limitation caused phosphate and nitrate deficiencies. The combination of activation (mediated by increased 3-PGA and reduced phosphate) and inhibition (mediated by the release of phosphate and nitrate) improved AGPase gene expression and enzyme activity (**Figures 3B**; **Supplementary Figure S10B**, and **3E**). AGPase expression and activity under LC treatment were 3.0× and 6.5× higher, respectively (**Supplementary Data S2**; **Figures 3A, B**). Most previous attempts to increase starch accumulation have focused only on enhancing AGPase gene expression instead of regulating its enzyme activity, which is possibly why these attempts were not highly successful (Cakir et al., 2019; Kang et al., 2013; Oiestad et al., 2016).

Furthermore, LC treatment increased the expression and activity of other important genes/enzymes, including granule-bound starch synthase (*GBSS*) and soluble starch synthase (*SSS*), and the expression of 1,4-alpha-glucan branching enzyme (*GBE*) (**Figures 3A, C**). Previous studies have shown that the genetic manipulation of any one of these genes usually has limited effects on starch content and does not increase the net starch yield (Ihemere et al., 2006; Stark et al., 1992; Sweetlove et al., 1996; Wang et al., 2007; Zeeman et al., 2010). Starch biosynthesis is a complex system that interconnects a wide variety of cellular processes and metabolic pathways (Geigenberger, 2011). Thus, it is very difficult to develop a comprehensive method to regulate this process. In our case, when the biomass increased by 6×, the starch content increased by 11×, and the starch yield increased by 76× within 10 days compared with that in the beginning stage (**Figures 1**, **2**). This is presumably because LC treatment significantly improved the activities of AGPase by transcriptional regulation and allosteric regulation, increased the gene expression level and activity of other key enzymes, and ultimately regulated the complex system.

#### 3.2.2 Distribution of carbon on lignocellulose and protein

The carbon skeletons of lignocellulose and protein are derived from photoassimilates, and their biosynthesis is strongly affected by carbon partitioning. The lignocellulose contents in duckweed are relatively low and are further reduced under LC treatment (**Figure 2A**). Initially, the cellulose, hemicellulose, and pectin contents were 8.0 ± 0.1%, 7.8 ± 0.1%, and 5.6 ± 0.3%, respectively (d.b.). After ten days of treatment, these values decreased to 1.1 ± 0.2%, 4.1 ± 0.1%, and 1.0 ± 0.0% (d.b.) (**Figure 2A**), corresponding to reductions of 86.3%, 47.4%, and 82.1%, respectively. The expression level of sucrose synthase (SUSY), an enzyme involved in the formation of UDP-glucose from sucrose to cellulose and hemicellulose, was significantly downregulated (**Supplementary Figure S12**; **Supplementary Data S7**). With respect to cellulose degradation, the expression of genes in glycoside hydrolase family 9 (cellulase) was significantly upregulated more than 5x (**Supplementary Data S8**). These changes might contribute to the reduction in cellulose and hemicellulose contents.

Lignin, a complex phenol polymer that is difficult to degrade, is the main obstacle for biomass utilization. The lignin content in duckweed decreased by 81.0% (**Figure 2A**), from 5.8 ± 0.1 to 1.1 ± 0.1%, after 10 days of LC treatment. Expression of the gene encoding laccase, the key enzyme for monolignol polymerization and crosslinking, was extremely low (FPKM values < 20) (**Supplementary Figure S12**; **Supplementary Data S9**), providing a possible explanation for the low lignin content. Additionally, the lignocellulose composition was reduced to a very low level (6.3%), indicating a higher quality of the whole biomass (**Figure 2A**).

Notably, the protein content of duckweed rapidly decreased from 30% to 4% (d.b.) under this treatment (**Figure 2A**). The total amount of protein remained essentially stable over 10 days due to the lack of a nitrogen supply (**Figure 2C**), whereas biomass, especially the starch content, still accumulated rapidly (**Figures 1B, C**). Nitrogen glutamine synthetase (*GS*), the key gene involved in nitrogen assimilation and recycling, plays an important role in increasing the nitrogen use efficiency (NUE, kg grain yield per kg N application) of crops and increasing cereal yield (Bernard and Habash, 2009; Xu et al., 2012). The expression level of *GS* was upregulated by 6.7×, and its enzyme activity was 7.4× greater than that of the control (**Supplementary Data S12** and **Supplementary Figure S11**). Previous studies have shown that nitrogen limitation significantly reduces the mRNA expression of *GS* and its enzyme activity and increases the starch content without increasing plant biomass or starch yield (Balotf et al., 2016; Hermans et al., 2006). However, in this study, both the plant biomass and starch yield increased, possibly by increasing the expression of *GS*, which strongly promoted the redistribution of ammonium and therefore increased protein reuse (**Supplementary Figure S13**). Thus, future research could focus on duckweed *GS*, especially its role in improving NUE.

The reduction in protein and lignocellulose contents in duckweed was consistent with the downregulated expression of relevant genes. This increase in starch content indicated that a large amount of photoassimilate flowed to starch synthesis under the LC treatment. Therefore, duckweed is an ideal model for studies of the regulation of nitrogen and carbon metabolism.

### 3.3 Gene numbers and regulation of carbon assimilation pathways

#### 3.3.1 Contraction of total gene numbers in carbon assimilation

We analyzed the genes of the carbon assimilation pathway in duckweed because of its strong starch accumulation ability. According to the results of whole-genome sequencing of *Landoltia punctata* 0202 (GenBank accession number: PRJNA546087) and the corresponding gene family analysis (**Supplementary Data S1**), the total number of genes involved in carbon assimilation, including starch metabolism, the Calvin cycle, and the Hatch−Slack cycle, was only 50. These values are significantly lower than those in Arabidopsis (64), rice (82), and maize (86) (**Table 1**). Under the LC treatment, in the carbon assimilation pathways, only the transcript levels of the key starch biosynthesis genes and the corresponding enzyme activities were upregulated (**Figures 3A-C** and **Supplementary Figure S3**). These starch biosynthesis genes work in a synergistically efficient way, hence enhancing starch formation. There was almost no change in gene expression in the Calvin cycle and no significant change in the activity of its key enzyme Rubisco (**Figures 3A** and **Supplementary Figure S8C**). In the Hatch-Slack cycle, some genes, such as *NADP-MDH*, *NADP-ME*, and *PEPC*, were also upregulated (**Figure 3A**), similar to the corresponding enzyme activities (**Supplementary Figures S8D,F**). LC treatment mainly increased starch biosynthesis and had a certain effect on the CO_2_ concentration but had no obvious effect on the Calvin cycle.

**Table 1:** Numbers of genes involved in starch metabolism.

Function	Enzyme	EC	KO	Lpu	Ath	Osa	Zma
Calvin cycle	Rubisco	4.1.1.39	K01602	5	4	6	2
	PGK	2.7.2.3	K00927	3	3	5	5
	GAPDH	1.2.1.12	K05298	1	3	2	5
	ALDO	4.1.2.13	K01623	3	8	8	7
	FBP	3.1.3.11	K03841	3	3	4	4
	Subtotal			15	21	25	23
Hatch-Slack cycle	PPDK	2.7.9.1	K01006	1	1	1	2
	PEPC	4.1.1.31	K01595	3	4	6	6
	NADP-MDH	1.1.1.82	K00051	1	1	1	1
	NADP-ME	1.1.1.40	K00029	2	5	6	8
	NAD-ME	1.1.1.39	K00028	2	2	2	2
	Subtotal			9	13	16	19
Starch synthesis	GPI	5.3.1.9	K01810	2	2	4	3
	PGM	5.4.2.2	K01835	2	3	2	3
	UGPase	2.7.7.9	K00963	2	2	3	2
	AGPase	2.7.7.27	K00975	4	6	6	7
	SSS	2.4.1.21	K00703	2	2	4	4
	GBSS	2.4.1.242	K13679	1	1	2	2
	GBE	2.4.1.18	K00700	4	3	3	5
	Subtotal			17	19	24	26
Starch degradation	amyA	3.2.1.1	K01176	1	2	8	6
	amyB	3.2.1.2	K01177	4	5	4	8
	MGAM	3.2.1.20	K01187	4	4	5	4
	Subtotal			9	11	17	18
Total				50	64	82	86

Lpu, *Landoltia punctata*; Ath, *Arabidopsis thaliana*; Osa, *Oryza sativa*; Zma, *Zea mays*.

Therefore, what is observed in duckweed contradicts the common knowledge that the more genes an organism possesses, the greater its function. Our results revealed that the changes in starch accumulation may have resulted from the regulation of the starch biosynthetic pathway rather than the number of gene copies.

#### 3.3.2 DNA methylation in gene regulation

DNA methylation, a conserved epigenetic modification, plays an important role in assisting in gene regulation and genome stability (Zhang et al., 2018). We studied the epigenome of DNA methylation in the same samples via transcriptome analysis. LC treatment decreased the DNA methylation level in the whole genome of duckweed from 12.9% to 11.2% (mC) at 24 h (**Supplementary Table S7**). More importantly, the DNA methylation levels of the *AGPase*, *SSS*, and *GBE* promoters were significantly reduced by 46.6, 32.2, and 63.6% (mCG), respectively, while their expression was significantly upregulated. Thus, in the starch biosynthetic pathway, DNA methylation in promoter regions is negatively correlated with gene expression (**Figures 3A, D**). In the Calvin cycle, although the DNA methylation level of the key genes’ promoters decreased, their expression levels did not change significantly. In the Hatch-Slack cycle, the DNA methylation level of the promoters of the same key genes was significantly increased, whereas their expression levels did not decrease. Notably, the expression level, DNA methylation level, and enzyme activity of NADP-MDH, one of the key genes in the Hatch-Slack cycle, increased significantly (**Figures 3A, D**; **Supplementary Figure S8E**). Previous studies have indicated that CO_2_ elevation can significantly increase the activity of NADP-MDH (Seth and Misra, 2014; Vu et al., 2006). It could be speculated that the increase in the NADP-MDH expression level under the LC treatment is due to the effect of elevated CO_2_.

Under LC treatment, DNA methylation did not affect the expression of key enzymes in the Calvin cycle or Hatch−Slack cycle and only played an essential role in the coordinated expression of key genes in the starch biosynthetic pathway.

#### 3.3.3 Transcription factors in the starch biosynthetic pathway

TFs also play important roles in regulating gene expression (Lai et al., 2019). By analyzing coexpression networks with key genes of the starch biosynthetic pathway, we predicted that some TFs were positively correlated. We found that multiple OBF1 genes, which are TFs of the bZIP family in duckweed, were positively correlated with all key genes involved in starch biosynthesis (*AGPase*, *SSS*, *GBSS*, and *GBE*) and presented high expression levels (FPKM increased from 140 to 400) (**Supplementary Data S16**). The bZIP TFs are key regulators of starch biosynthesis genes in rice, maize, and wheat and determine starch quality and quantity in the endosperm (Chen et al., 2016; Kumar et al., 2018; Singh et al., 2015; Wang et al., 2013).

The genes associated with carbon assimilation were all contracted (**Table 1**), but under the LC treatment, only starch biosynthesis ability was significantly enhanced, which was mutually confirmed by the changing trends at multiple levels (**Figures 1A, C**, **3**). Therefore, high starch yield can be obtained merely through the regulation of expression levels. This discovery deserves further consideration and research.

### 3.4 Sucrose biosynthesis and transportation in duckweed

#### 3.4.1 The “source–flow–sink” relation in plants

Sucrose biosynthesis and transportation are crucial for starch accumulation in plants (Julius et al., 2017). This process primarily includes photoassimilate synthesis in chloroplasts, transmembrane transport into the cytoplasm, sucrose biosynthesis, and long-distance transport of sucrose for subsequent conversion into starch in storage organs. The “source-flow-sink” relationship is highly related to crop yield. Our results demonstrated that the duckweed frond, a tissue similar to leaves, acts as a “sink” organ that accumulates stored starch (**Figure 1E**).

#### 3.4.2 Conversion of “sources” to “sinks” in duckweed chloroplasts

**Figure 4:**
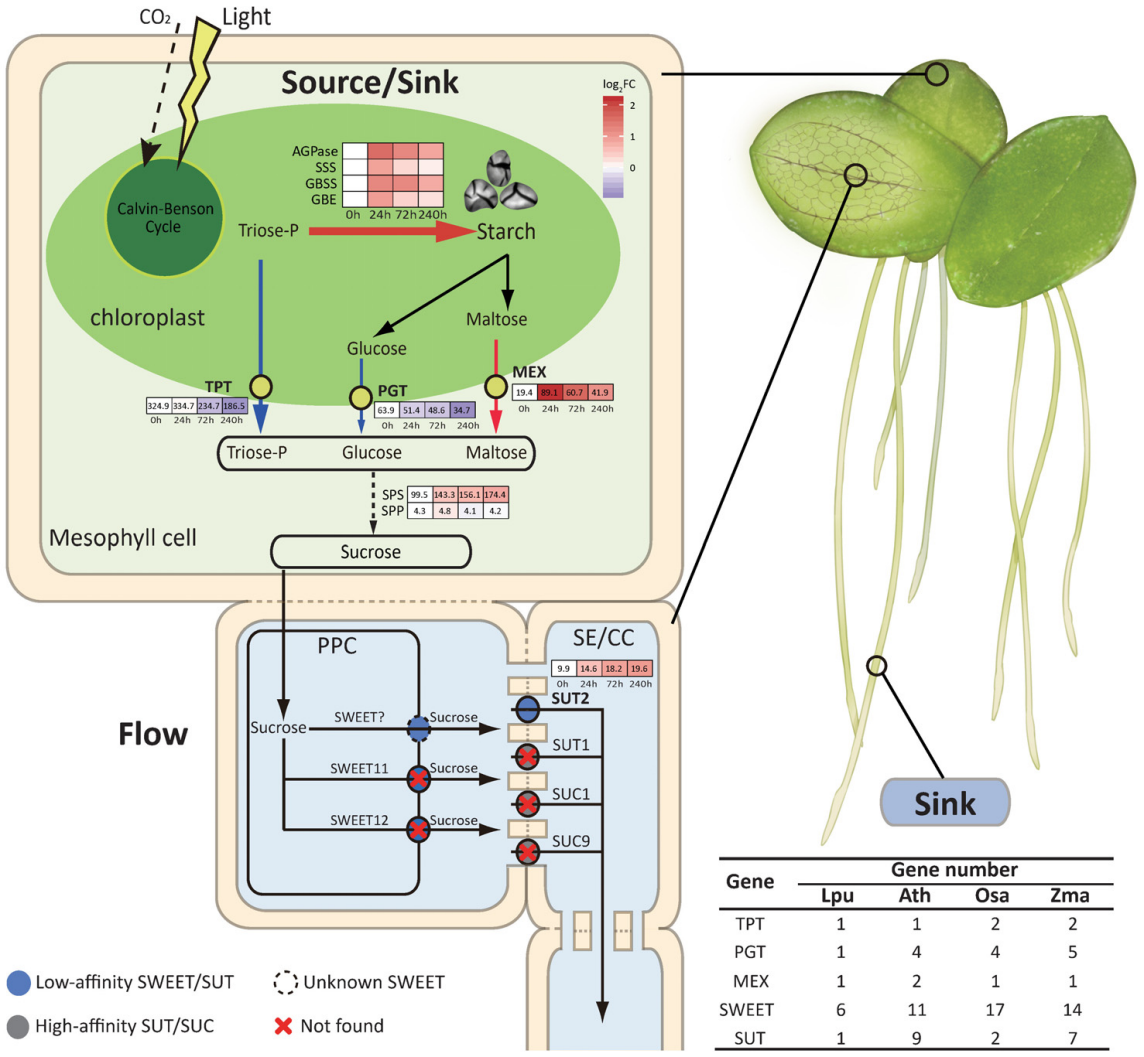
Sugar biosynthesis and transportation in *Landoltia punctata* under the LC treatment. The source, flow, and sink in *Landoltia punctata* are represented in light green, light yellow, and light purple, respectively. The heatmaps show the expression profiles of genes involved in starch synthesis and the transport of triose-P, glucose, maltose, and sucrose. The numbers in the boxes are the FPKM values. The color of the boxes indicates the log_2_FC, where the FC represents the fold change in the expression level compared with that at 0 d. The thickness and length of the arrows represent the strength of sugar flux. Red, upregulated expression; blue, downregulated expression; numbers in brackets, gene numbers of transporter proteins. SE/CC, sieve element/companion cell complex.

The transportation of photoassimilates from chloroplasts relies on the triose phosphate/phosphate translocator (*TPT*), glucose transporter (*PGT*), and maltose transporter (*MEX*). Correspondingly, deletions and mutations of *TPT*, *PGT*, and *MEX* lead to starch accumulation in the chloroplast (Cho et al., 2011; Jang et al., 2015; Walters et al., 2004). Duckweed has a markedly contracted number of genes involved in the transportation of photoassimilates. TPT, PGT, and MEX were reduced to only one copy each (**Figure 4**; **Supplementary Data S3**). Furthermore, the expression levels of both *TPT* and *PGT* were significantly downregulated under the LC treatment. Notably, *TPT*, the major export transporter of photoassimilates from chloroplasts, decreased by 42.6% (**Figure 4**). In duckweed mesophyll cells, LC treatment significantly increased the expression of *AGPase* by 3.0× in the chloroplast but suppressed the export of plastidial triose phosphorate and glucose to the cytosol, resulting in the hyperaccumulation of starch in the chloroplast (**Figures 1E**, **3E**, **4**). The subcellular localization of starch granules, AGPase, TPT, PGT, and MEX also confirmed the transition of chloroplast function from being the “source” to the “sink” in duckweed (**Figures 1**; **Supplementary Figure S6**). The source and sink are thus spatially organized together in the chloroplast, in stark contrast to other crops. This treatment allows duckweed chloroplasts to be highly efficient at forming and storing starch.

#### 3.4.3 Weak “flow” in duckweed

The volume of the “flow” in duckweed is affected by the quantity of sucrose and the efficiency of the transporter. The sucrose content in duckweed is normally < 1.1 mg g^-1^ FW, which is much lower than that in corn and rice (**Supplementary Figure S18**). Sucrose synthesis is regulated by two main enzymes, sucrose phosphate synthase (SPS) and sucrose phosphate phosphatase (SPP). SPS is a reversible rate-limiting enzyme that catalyzes the synthesis of sucrose-6P using UDP-glucose and fructose-6P and reversely catalyzes the degradation of sucrose-6P (Huber and Huber, 1996). The number of *SPS* genes (4) in duckweed was lower than that in rice, corn, and cassava (**Supplementary Data S16A**). *SPP*, more importantly, is present in only one copy, with a very low expression level (FPKM values <5), resulting in the accumulation of the substrate sucrose-6P and subsequently promoting the reverse catalysis of sucrose-6P degradation. Under LC treatment, the expression level of *SPS*, whose FPKM value was already greater than 84 at the beginning (0 h), increased significantly (Log_2_FC =1.26 for 240 h vs. 0 h), leading to further enhancement of the degradation of sucrose-6P and ultimately resulting in a very low sucrose concentration in the cytoplasm (**Supplementary Data S16B**; **Supplementary Figure S18**). Thus, duckweed has an extremely weak ability to synthesize sucrose and has a low sucrose content in its cell cytoplasm, that is, a low volume of “flow”.

Sucrose transporters (*SUT*s) and hexose and sucrose transporters (*SWEET*s) are responsible for the long-distance transport of sucrose to nonphotosynthetic organs. Among them, SUTs are the most important transporters. Duckweed possesses only one *SUT* gene (*LpSUT*) and 6 types of *SWEET* genes (**Figure 4**; **Supplementary Data S4** and **Supplementary Data S6**). Compared with Arabidopsis, which possesses 9 *SUT* genes, 4 of which have high affinity, the SUT protein in duckweed might have low affinity. The extended N-terminus of LpSUT has a lower affinity for sucrose, which is highly similar to SUT2 in Arabidopsis, the sucrose transporter with the lowest affinity (Schulze et al., 2000) (**Supplementary Figure S17**). We also observed a very low expression level of *LpSUT* (FPKM values 9.9–19.6) (**Supplementary Data S6**). On the other hand, duckweed contains many fewer copies of *SWEET* genes than do Arabidopsis and rice. Duckweed also lacks homologues of *SWEET11* and *SWEET12*, the key sucrose efflux transporters (Chen et al., 2012) (**Supplementary Data S4**). At the transcriptional level, the expression of the *SWEET* genes in duckweed was also very low (FPKM values <30) (**Supplementary Data S5**). Therefore, the number of SUT and SWEET genes and their expression levels showed a weak “flow” ability in the plants. Impressively, the number of genes regulating sucrose transportation was reduced, and only those with low affinity remained. Owing to the reduced gene number and weakened protein activities, sucrose metabolism was markedly suppressed in terms of synthesis and transportation.

The low sucrose concentration, low sucrose synthesis, and low transport capacity resulted in a weak “flow” in duckweed. LC treatment strongly stimulated starch accumulation in the chloroplast and further reduced its sugar flow ability, turning the “source” frond into a “sink” organ (**Figure 4**). Therefore, duckweed is an unusual and interesting system in which sources and sinks are spatially organized together, unlike the interdependent compartmentation of sinks and sources in other higher plants.

### 3.5 Prospects for the application of duckweed

In the past 50 years, the wide application of green revolution technology has resulted in extraordinary achievements in staple crop production worldwide, especially with the sharp increase in the crop harvest index (grain-straw ratio) from 0.3 to 0.5. Currently, further increasing the harvest index is very difficult because only certain parts of the crop can be harvested. In contrast, the harvest index of duckweed is nearly 1.0 because of its high starch content and low lignocellulose content (~5.8%), especially its lignin content (~1.1%) (**Figure 2**). Thus, whole duckweed can be harvested and used completely. Furthermore, unlike the reproductive growth of cereal crops, the production ability of duckweed depends on vegetative growth and avoids the time-consuming phase of organ development and differentiation, as well as the fragile stage of sexual reproduction (such as flowering, pollination, etc.) Therefore, the starch productivity of duckweed is considerably greater and more stable than that of staple crops.

The extensive use of green revolution varieties (GRVs) has led to excessive consumption and waste of fertilizer, which has thus resulted in serious environmental problems. Since GRV lodging resistance is enhanced by relative insensitivity to nitrogen, GRVs are associated with reduced NUE. Our results demonstrated that duckweed efficiently assimilated carbon under the LC treatment without being supplied with any exogenous nitrogen or phosphorus. The NUE of duckweed reached 144.4 kg biomass kg^-1^ N, which was much greater than those of maize, rice, and wheat. In the post-green revolution era, an important research and development direction of agriculture has been to improve the NUE of crops, and starch production using duckweed is a good choice. Moreover, the LC treatment increased the absorption of CO_2_ by duckweed, reducing the emission of greenhouse gas.

The starch content of other duckweed species, such as *Spirodela polyrhiza* and *Lemna minor*, can also reach 45.68-57.23% when this technology is used, confirming its universal applicability for efficient starch production in duckweed (**Supplementary Figure S19**). Moreover, a pilot scale was carried out beside Dianchi Lake, southwest of Kunming (E 102°47′, N 24°51′). The starch content of the cultivated duckweed reached 45.9 ± 3.5% (d.b.) within 4 days, and the starch productivity reached 36.5 t ha^-1^ y^-1^ (**Supplementary Table S2**). Thus, LC treatment has great potential in practical applications.

## 4 Conclusions

This study is the first report of a simple and environmentally friendly technology for starch production using duckweed. The starch content and productivity reached 72.2% (dry basis) and 10.4 g m^-2^ d^-1^, respectively, in 10 days, equivalent to a yield of 38.0 t ha^-1^ y^-1^ under nutrient limitation and CO_2_ elevation treatments. Furthermore, the relevant mechanism of high starch accumulation in duckweed was investigated. The results revealed that the regulation of DNA methylation and transcription factors, as well as the significantly upregulated transcription levels and increased enzyme activities of key genes involved in starch biosynthesis, caused high starch accumulation in duckweed. This technology is easy to operate and viable for achieving agricultural industrialization. This work demonstrated that duckweed could be a next-generation starch crop and an ideal model plant for starch metabolism research.

## Data Availability

All data are available in the manuscript, the supplements, or publicly accessible repositories. The raw reads from whole-genome sequencing of Landoltia punctata 0202 have been deposited at NCBI under BioProject ID: PRJNA546087. The whole-genome data of the other species used in this study are available in the **Supplementary Materials.** All the transcriptomes have been uploaded to NCBI under BioProject ID PRJNA672224. All epigenetic data have been deposited in NCBI under BioProject ID: PRJNA673253. The duckweed samples are available from the Duckweed Resource Bank at the Chengdu Institute of Biology, Chinese Academy of Sciences.
