# Supplementary material for: Duckweed: a starch-hyperaccumulating plant under cultivation with a combination of nutrient limitation and elevated CO2
Source: Front Plant Sci. 2025 Feb 10;16:1531849. doi: 10.3389/fpls.2025.1531849 (PMC11847889; doi:10.3389/fpls.2025.1531849)
Supplement: Supplementary file 2 [file DataSheet2.docx]

Supporting Information

**Duckweed: a starch-hyperaccumulating plant under cultivation with a combination of nutrient limitation and elevated CO_2_**

Ling Guo^1, 2†^, Yang Fang^1†^, Songhu Wang^1^, Yao Xiao^3^, Yanqiang Ding^1^, Yanling Jin^1^, Xueping Tian^1^, Anping Du^1^, Zhihua Liao^4^, Kaize He^1^, Shuang Chen^1^, Yonggui Zhao^5^, Li Tan^1^, Zhuolin Yi^1^, Yuqing Che^1^, Lanchai Chen^1^, Jinmeng Li^1^, Leyi Zhao^6^, Peng Zhang^7^, Zhengbiao Gu^8^, Fangyuan Zhang^4^, Yan Hong^8^, Qing Zhang^9^, Hai Zhao^1^*

^1^ CAS Key Laboratory of Environmental and Applied Microbiology, Environmental Microbiology Key Laboratory of Sichuan Province, National Engineering and Research Center for Natural Medicines, Chengdu Institute of Biology, Chinese Academy of Sciences, Chengdu 610041, China.

^2^ Department of Pediatrics, Children Hematological Oncology and Birth Defects Laboratory, The Affiliated Hospital of Southwest Medical University, Sichuan Clinical Research Center for Birth Defects, Southwest Medical University, Luzhou 646000, China.

^3^ Analytical and Testing Center, Sichuan University of Science and Engineering, Zigong 643000, China.

^4^ Key Laboratory of Eco-environments in Three Gorges Reservoir Region (Ministry of Education), SWU-TAAHC Medicinal Plant Joint R&D Centre, School of Life Sciences, Southwest University, Chongqing 400715, China.

^5^ School of Ecology and Environmental Sciences & Yunnan Key Laboratory for Plateau Mountain Ecology and Restoration of Degraded Environments, Yunnan University, Kunming 650091, China.

^6^ Pitzer College, California, 91711, United States.

^7^ National Key Laboratory of Plant Molecular Genetics, CAS Center for Excellence in Molecular Plant Sciences, Chinese Academy of Sciences, Shanghai 200032, China.

^8^ School of Food Science & Technology, Jiangnan University, Wuxi 214122, China.

^9^ College of Food and Bioengineering, Xihua University, Chengdu 610039, China.

^†^These authors contributed equally to this work.

*Corresponding author. Email: [zhaohai@cib.ac.cn](mailto:zhaohai@cib.ac.cn)

Supplementary Text

1 Transcriptomes

1.1 RNA Sequencing

7.2 Gb raw bases were acquired for each duckweed sample (Table S2), with approximately 7 Gb of clean bases. The Q20s of bases were above 97.2%, indicating that the sequences could be used for further analysis.

1.2 Validation of RNA Sequencing

Twenty DEGs were selected to validate RNA sequencing by qRT-PCR. Among the selected DEGs, the expression patterns of 19 genes in qRT-PCR were similar to those in RNA sequencing (Figure S4), suggesting an overall consistency between RNA sequencing data and qRT-PCR results. Therefore, the RNA-sequencing in this study was reliable.

2 Quantification of key genes expression

Expression of key genes involved in CO_2_ fixation, carbon concentration, and starch synthesis (*PEPC*, *Rubisco*, *UGPase*, *AGPase*, *SSS*, and *GBSS*) was quantified by qRT-PCR. Results from qRT-PCR of key genes (Figure S5) were similar to those of FPKM values from RNA sequencing, showing an increase of the expression level of most key genes induced by LC treatment.

3 Subcellular localizations of enzymes and translocators

We investigated the subcellular locations of AGPase (10015507), GBSS (10021837), SSS (10010699), UGPase (10020333), plastidic glucose translocator (PGT), maltose transporter (MEX), triose phosphate/phosphate translocator (TPT), sucrose transporters (SUT), hexose and sucrose transporter SWEET1 (10012584), and SWEET7B (10002893) in *Landoltia punctata*. To this end, GFP were fused to the N-terminus of SUT protein and to the C-terminus of the rest of the proteins. These proteins were then assessed for their subcellular localization in duckweed protoplast cells.

Transient expression of AGPase-, GBSS-, and SSS-GFP fusion protein in protoplasts revealed their co-localization with chlorophyll, which confirms their presence in chloroplasts and their participation in starch biosynthesis (Figure S6 A-C). Transient expressions of PGT-, MEX-, TPT-GFP fusion proteins also confirmed the transporters localized mainly on chloroplasts. Thus, the three transporters in chloroplasts might work for sugar transport (Figure S6 D-F) (Smith et al., 2005). Specially, transient expression of UGPase-GFP fusion protein showed it localized mainly in cytoplasm, suggesting UGPase as a cytosolic type in *Landoltia punctata* (Figure S6 G).

Transient expression of SWEET1- and SWEET7B-GFP fusion protein in protoplasts revealed that they localized mainly on cell membrane, confirming SWEET transporters’ ability to transport sugar on cell membranes (Figure S7 A-B). The transient expression of sucrose transporter (SUT) in protoplasts showed that GFP-SUT fluorescence is mainly retained in intracellular structures, similar to the SISUT2 subcellular localization in tomato (Figure S7 C) (Krugel and Kuhn, 2013). This suggests it might lose its function as a sucrose transporter in *Landoltia punctata*.

4 DNA methylation analysis

WGBS libraries were subjected to pair-end sequencing using Illumina HiSeq X Ten platform. Filtered high quality reads were aligned against the genome of *Landoltia punctata* (Table S5). Identification of methylated cytosine positions for each sample was performed independently.

The global methylation level of *Landoltia punctata* ranged from 11.2% to 13.0%. Primarily, methylation level involving CG sequences was more than 70% (Table S6 and Figure S20). The methylation levels of different genome regions (exons, introns, and 2 kb-upstream and -downstream of genes) decreased under LC treatment (Figure S21). In particular, methylation levels of CG sequences in exons, introns, and the 2 kb-upstream of genes decreased dramatically and remained relatively low during the 10-day cultivation under LC treatment (Figure S22).

5 Universal applicability of LC treatment

Two of other duckweed species, *Spirodela polyrhiza* and *Lemna minor*, also accumulated starch content to over 45% under LC treatment (Figure S19). In the pilot-scale test when duckweed was harvested every 4 days, starch content and yield reached 45.9 ± 3.5 % and 10.0 ± 1.4 g m^-2^ d^-1^, equivalent to annual starch yield of approximately 36.5 t ha^-1^ (Table S7). This indicated the universal applicability of LC treatment for efficient starch production in duckweeds.

**Results**

**Starch accumulation in Duckweed fronds**

*Landoltia punctata 0202* was previously identified to be a useful duckweed ecotype with great potential for starch accumulation. In this study, the novel methods, combining the nutrient limitation (“L” in short) and the elevated CO_2_ concentration (“C” in short), were established to induce starch production. Biomass of duckweeds reached 144.7 g m^-2^ in 10-day LC treatments, and that of control was only 100.8 g m^-2^, indicating that LC treatment markedly enhanced biomass accumulation of duckweed (Fig. 1B). The starch contents raised from 6.5% to 72.2% (dry weight, DW) under LC treatments for 10 days (Fig. 1A), and net amount of 104 g starch was produced per square meter (Fig. 1C).

**Carbon partitioning under LC treatments**

Quantitative measurements indicated that the contents of most determined components except starch were decreased significantly after LC treatments (Fig. 2A). Especially, the protein content dropped from 30% to 4% (DW) (Fig. 2A). Duckweeds contained relatively low contents of the lignocellulose components (cellulose, hemicellulose, pectin, and lignin) and LC treatments reduced their accumulation to even lower levels (Fig. 2A).

**Copy numbers of starch metabolism and sugar transportation genes in the genome**

We have sequenced the whole genome of *Landoltia punctata* 0202. We *de novo* assembled 48,966 scaffolds (L50 = 4.0 Mb) covering 422.4 Mb of its genome. Annotation revealed 19,692 nuclear-encoded protein-coding genes, of which 86.0% was supported by transcriptome data (Tables S3 and Data S1).

We assembled a high-quality genome sequence of *Landoltia punctata* 0202 (GeneBank accession number). The copy numbers of Calvin cycle, Hatch-Slack cycle, starch metabolism and sugar transportation genes are significantly contracted compared to other higher plants, and the total number (60) of abovementioned genes in duckweed is much less than that in Arabidopsis (97), rice (102), and maize (115) (Table 1).

**Expression levels and enzyme activities of starch metabolism pathway**

Transcriptome analysis was performed on the samples of LC treatments. The results of quantitative reverse transcription PCR (qRT-PCR) showed that most of the validated genes showed similar expression patterns with the results of transcriptome analysis. (Fig. S4). Transcripts of the core starch biosynthesis genes are almost entirely up-regulated (Fig. 3A and Fig. S5). Some genes involved in Hatch-Slack cycle also obviously increased (Fig. 3A) and the corresponding enzyme activities also showed the similar patterns (Fig. S8). These results suggested that LC treatments enhanced the starch biosynthesis and the carbon concentration, but had no obvious effect on Calvin Cycle and starch degradation.

**DNA methylation under LC treatments**

To gain insight into the LC-induced transcriptional modulation, we characterized the epigenome of DNA methylation in the same samples of transcriptome analysis. The results showed that LC treatments decreased the level of genome-wide DNA methylation from 12.9% to 11.2% (mC) in 24h (Table S7). Especially, LC treatments significantly reduced the DNA methylation levels in the promoters of the genes encoding key enzymes in starch biosynthesis. Bisulfite sequencing PCR analysis also validated the reduction in DNA methylation levels in promoters of these genes.

**Characterization of the genes involved in sucrose biosynthesis and transportation**

Sucrose biosynthesis and transportation is crucial for starch accumulation. It primarily includes transportation of photoassimilates from chloroplast to cytosol in mesophyll cell, sucrose biosynthesis and long-distance transportation of sucrose. The former involves in triose phosphate/phosphate translocator (*TPT*), glucose transporter (*PGT*), and maltose transporter (*MEX*). In the genome of *Landoltia punctata*, there is only 1 copy of *TPT*, *PGT*, and *MEX*, respectively (Table 1 and Data S3). The expression of *TPT* and *PGT* was suppressed (Fig. 4).

After SUSY catalysis, the synthesized sucrose is transported to non-photosynthetic organs with the aid of sucrose transporters (*SUT*s) and hexose and sucrose transporter (*SWEET*s) which is called “flow”. *Landoltia punctata* possesses only 1 *SUT* gene (named as *LpSUT*) with very low expression level (FPKM values 9.9-19.6) (Table 1 and Fig. 4), and 10 *SWEET* genes also have low expression level (FPKM values < 30) (Data S4 and S5).


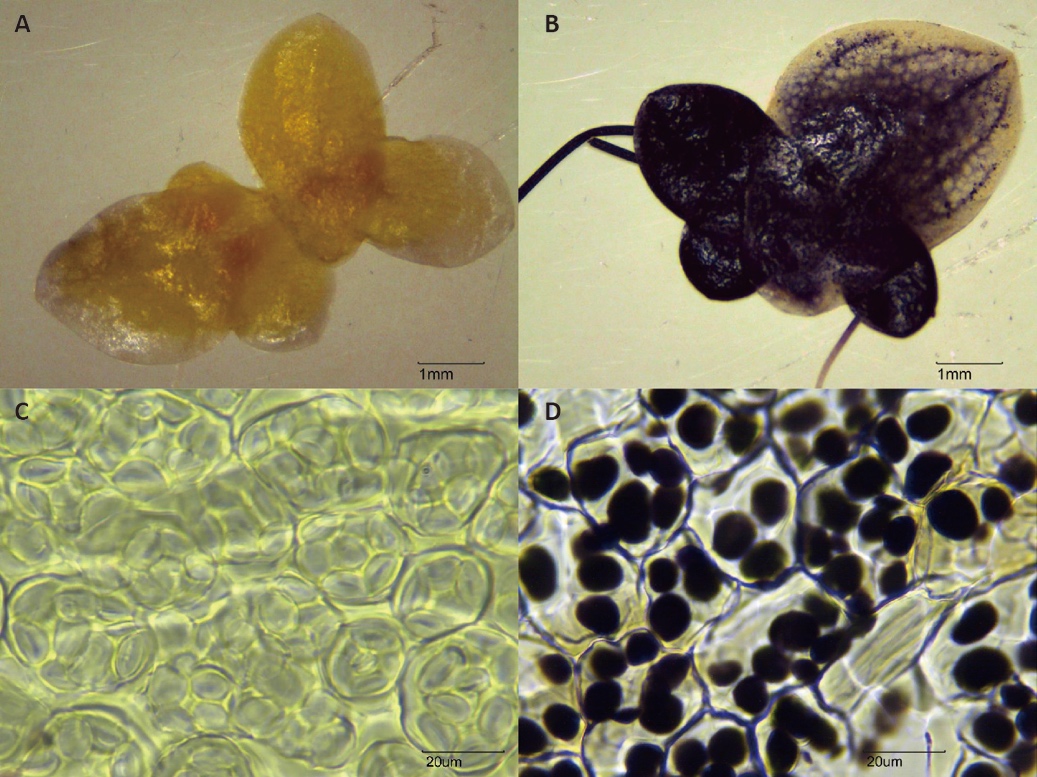


Figure S1.

Iodine staining of starch granules in *Landoltia punctata*.

**A and C**, iodine staining of the whole frond (A) and semithin sections (C) in the control treatment. The fronds were cultivated in 1/5 Hoagland media for 72 h.

**B and D**, iodine staining of the whole frond (B) and semithin sections (D) in the LC treatment. The fronds were cultivated under conditions of nutrient limitation and elevated CO_2_ level (2500±100 ppm) for 72 h.

Bars, 1 mm in A and B; 20 μm in C and D.

Figure S2.

Changes in moisture content of *Landoltia punctata* under LC treatment.

LC, cultivated under conditions of nutrient limitation and elevated CO_2_ level (2500±100 ppm). Letters indicate significant differences among time points, tested by one way ANOVA following Tukey-Kramer test (p<0.05).


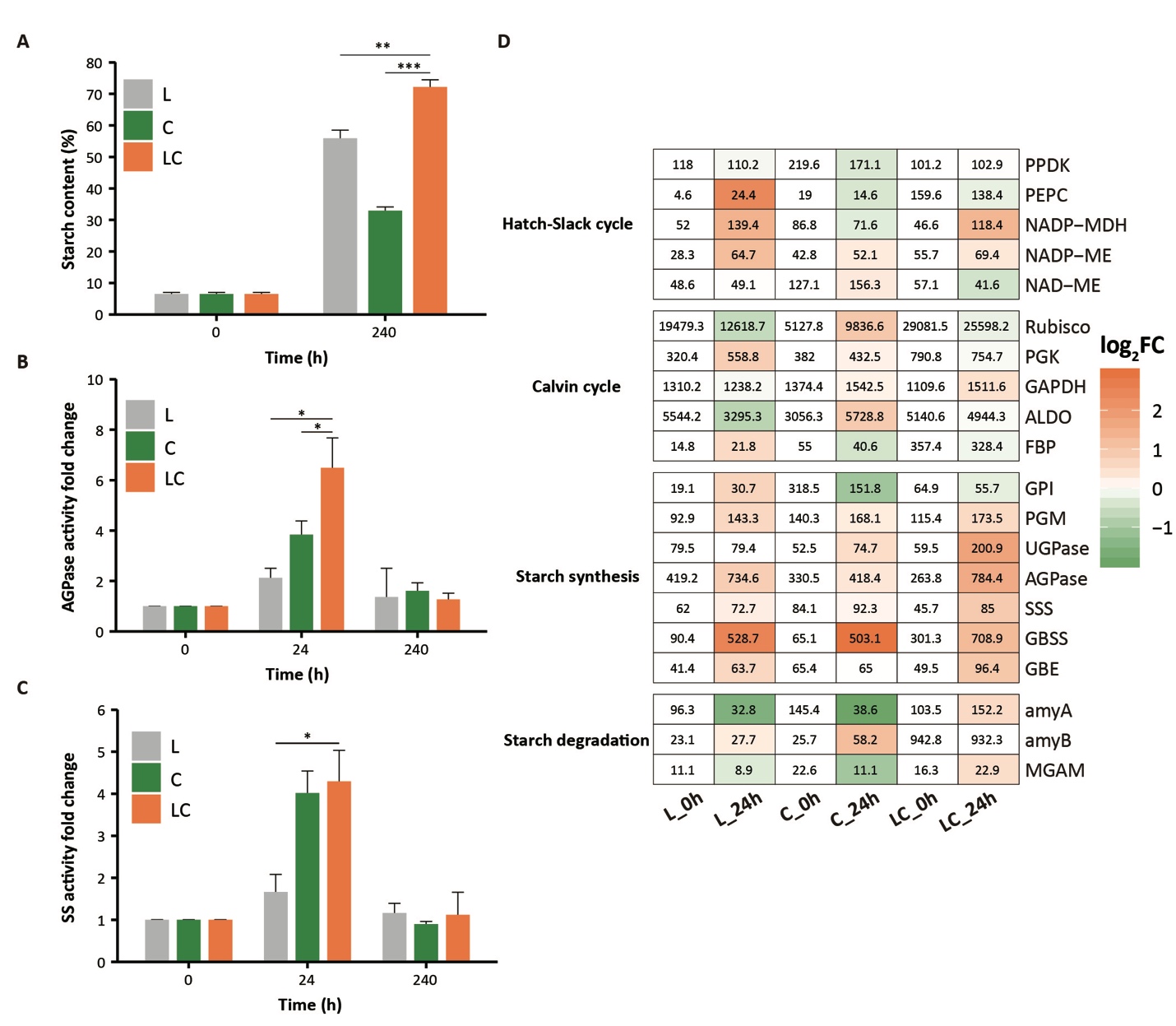


Figure S3.

Starch accumulation and biosynthesis in *Landoltia punctata* under different conditions.

**A**, Starch content.

**B**, Fold change of AGPase activity compared with 0 h.

**C**, Fold change of SS activity compared with 0 h.

**D**, Expression of genes involved in starch biosynthesis under various conditions. Numbers indicate the FPKM values. Colors indicate log_2_FC comparisons of expression values at 24 h and 0 h.

L, cultivated under condition of nutrition limitation;

C, cultivated under condition of elevated CO_2_ level (2500±100 ppm);

LC, cultivated under conditions of nutrient limitation and elevated CO_2_ level (2500±100 ppm).

Error bars are standard deviations measured from three independent cultures. Asterisk indicates statistically significant difference between treatment group and control in the same assay conditions (Student’s *t*-test). *, P<0.05; **, P<0.01; ***, P<0.001.


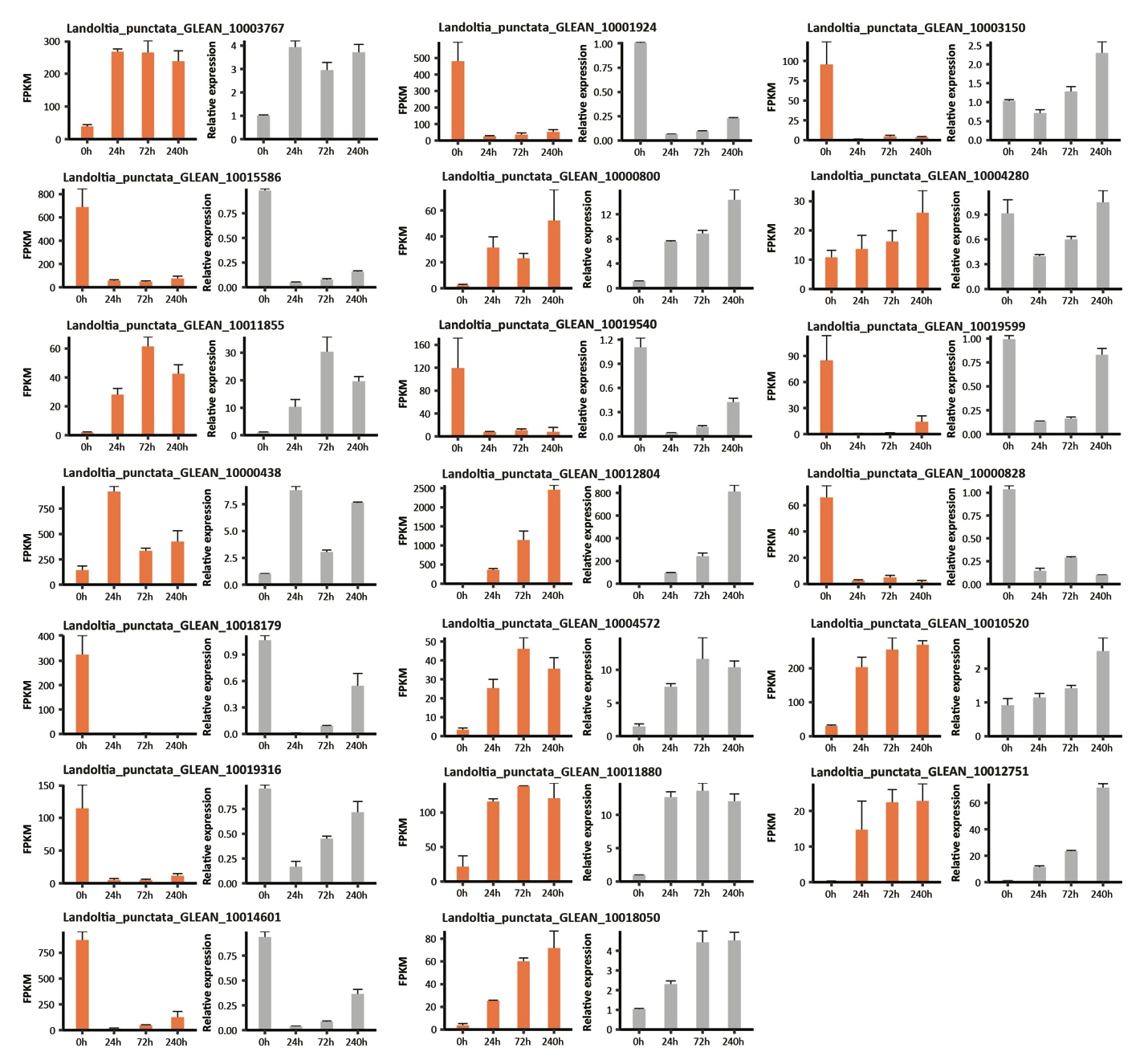


Figure S4.

Validation of RNA-Sequencing by qRT-PCR

For each pair of graphs, the left exhibits results from RNA sequencing while the right exhibits qRT-PCR data. Y-axis of RNA sequencing graphs represent FPKM value, while that of qRT-PCR graphs represent relative expression level, the expression level of the target gene normalized to that of internal control *Actin*.


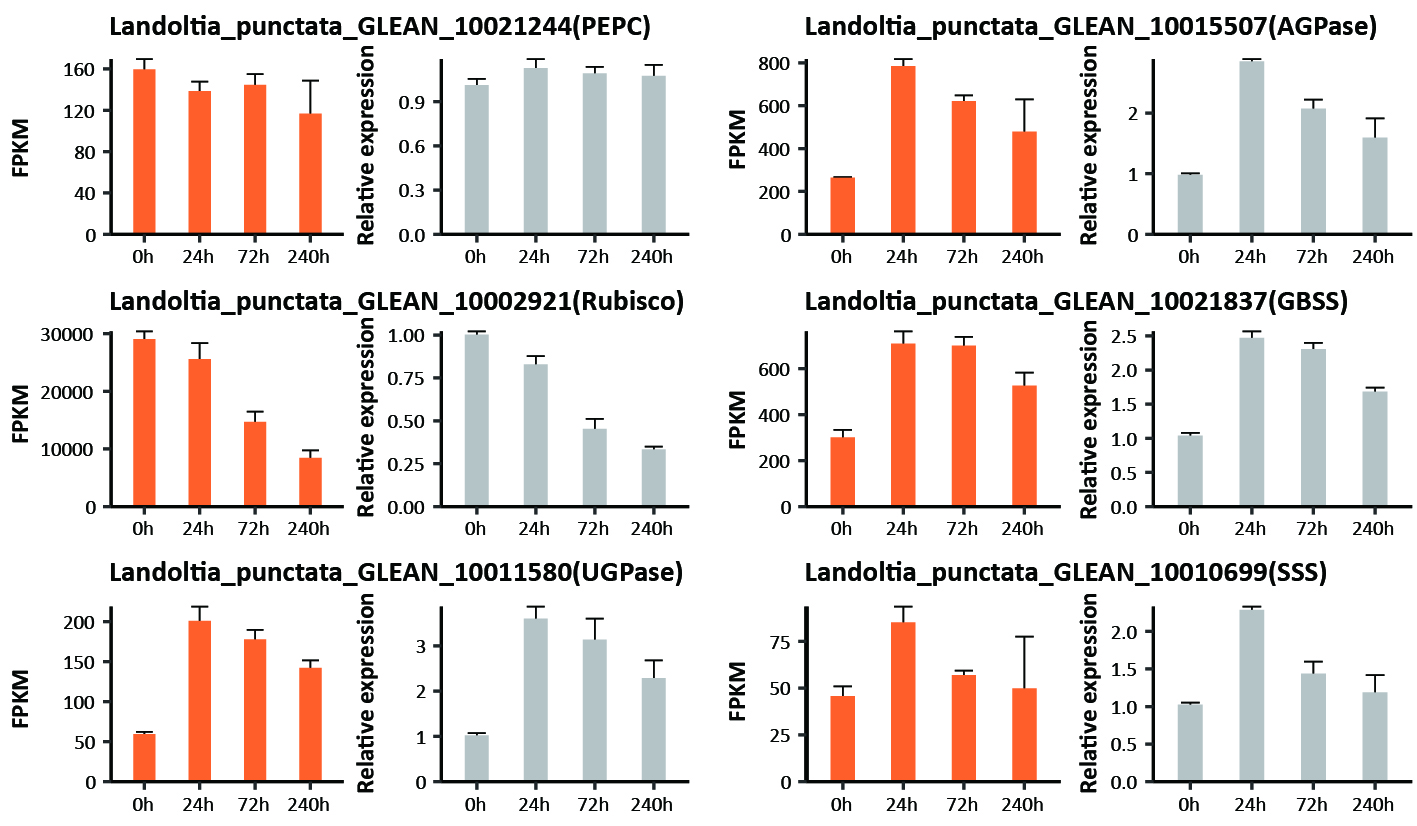


Figure S5.

Expression levels of key genes involved in CO_2_ fixation, carbon concentration, and starch synthesis as analyzed by qRT-PCR.

For each pair of graphs, the left exhibits result from RNA sequencing while the right exhibits qRT-PCR data. Y-axis of RNA sequencing graphs represent FPKM value, while that of qRT-PCR graphs represent relative expression level, the expression level of the target gene normalized to that of internal control *Actin*.


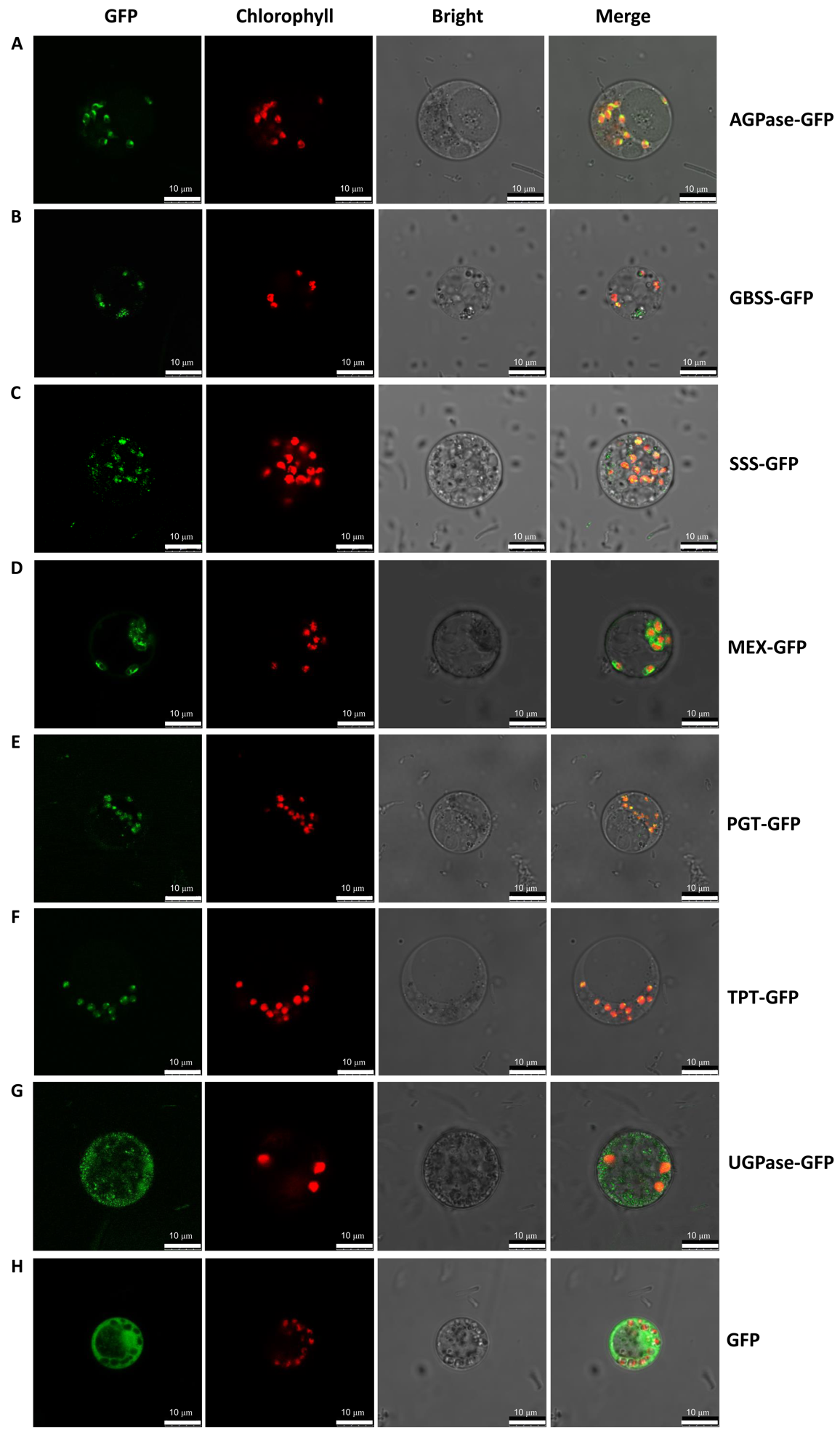


Figure S6.

Confocal laser scanning microscopy images of duckweed (*Lemna minor*) protoplast cells showing Subcellular localization of AGPase (10015507), GBSS (10021837), SSS (10010699), MEX, PGT, TPT, and UGPase (10020333) proteins.

**A-G**, target proteins with GPF-fused C-terminus

**H**, GFP protein, used as control

The green fluorescence signal indicated the target proteins or GFP protein. The red ﬂuorescence signal indicated the autoﬂuorescence of chloroplasts.


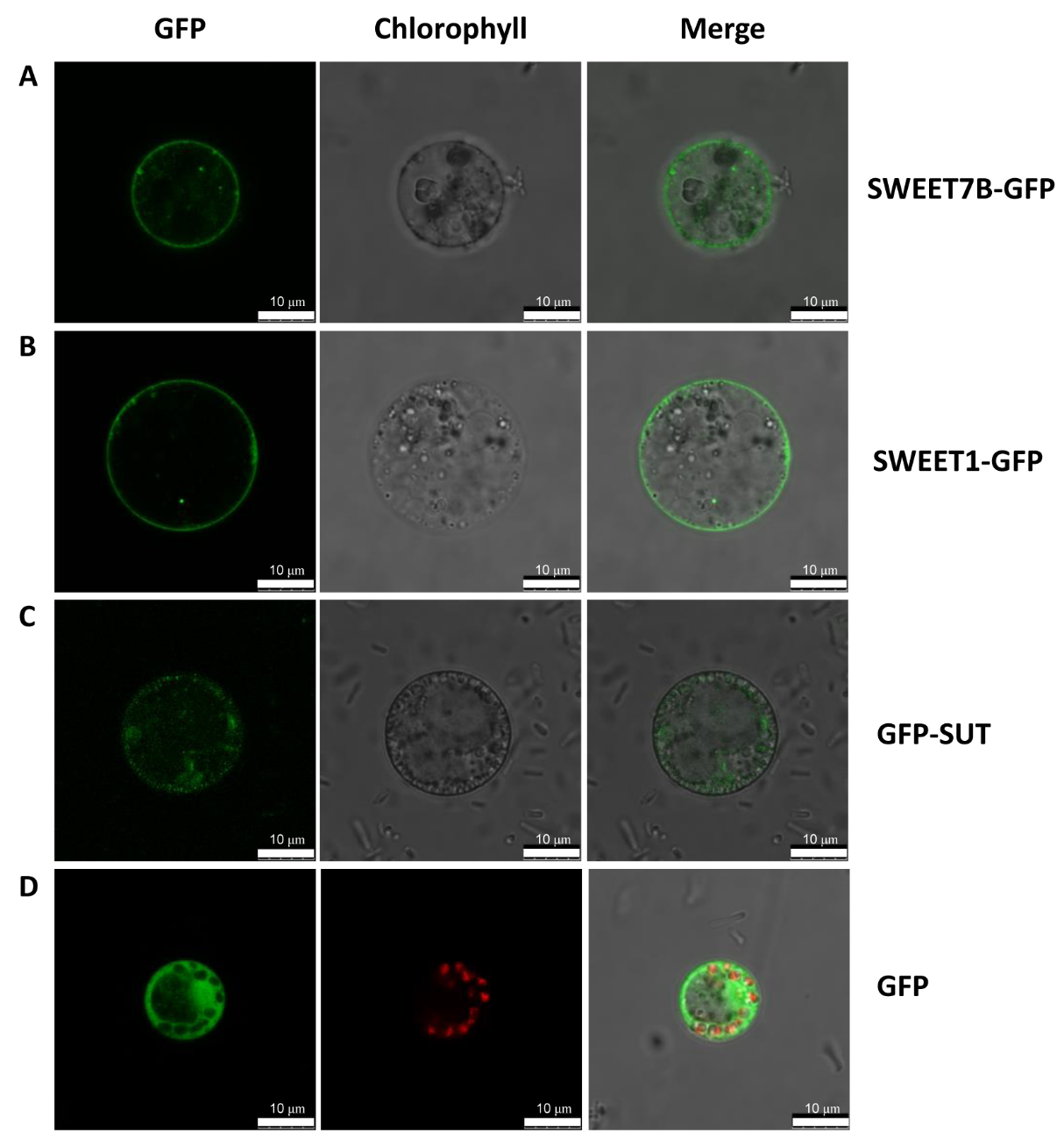


Figure S7.

Confocal laser scanning microscopy images of duckweed (*Lemna minor*) protoplast cells showing subcellular localization of SWEET (SWEET7B, 10002893; SWEET1, 10012584) and SUT proteins.

**A and B**, SWEET proteins with GPF-fused C-terminus

**C**, SUT protein with GFP-fused N-terminus

**D**, GFP protein, used as control

The green fluorescence signal indicated the fusion proteins. The red ﬂuorescence signal indicated the autoﬂuorescence of chloroplasts.


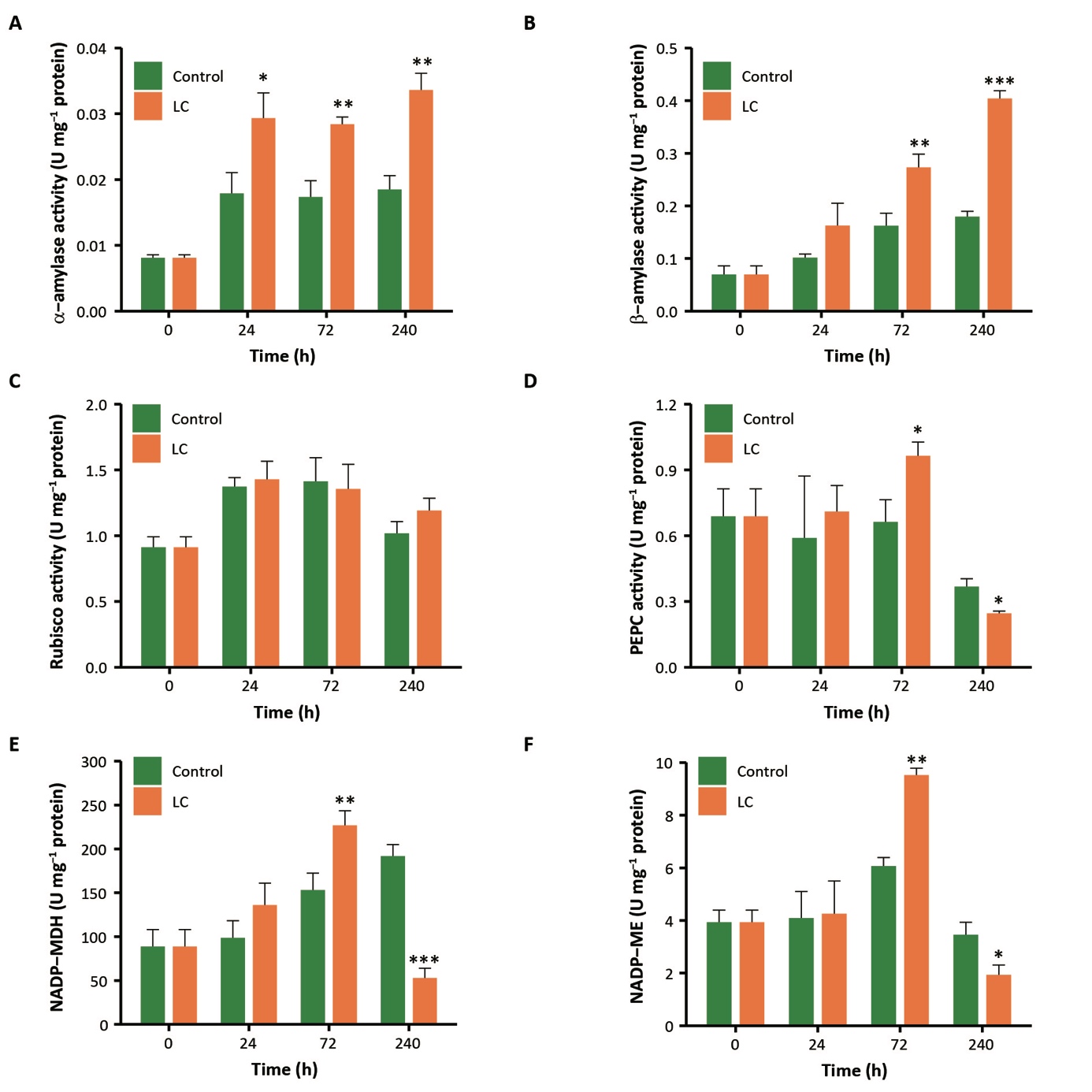


Figure S8.

Activities of (**A**) α-amylase, (**B**) β-amylase, (**C**) Rubisco, (**D**) PEPC, (**E**) NADP-MDH, and (**F**) NADP-ME in *Landoltia punctata*.

LC, cultivated under conditions of nutrient limitation and elevated CO_2_ level (2500±100 ppm). Control, cultivated in 1/5 Hoagland medium. Rubisco, ribulose-bisphosphate carboxylase; PEPC, phosphoenolpyruvate carboxylase; NADP-MDH, malate dehydrogenase (NADP^+^); NADP-ME, malate dehydrogenase (NADP^+^). Error bars show standard deviations measured from three independent cultures. Asterisk indicates statistically significant difference between treatment group and control in the same assay conditions (Student’s *t*-test). *, P<0.05; **, P<0.01; ***, P<0.001.


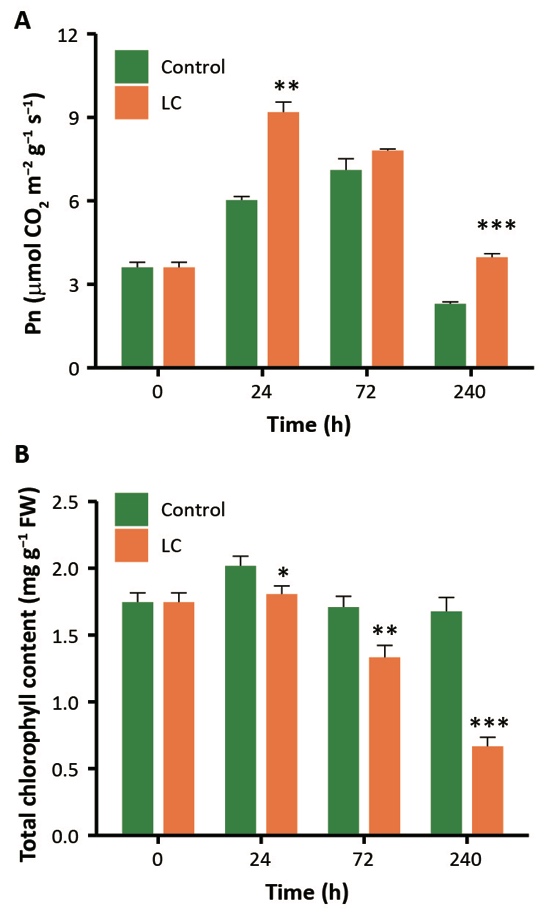


Figure S9.

Net photosynthetic rate (**A**) and total chlorophyll content (**B**) of *Landoltia punctata*.

LC*,* cultivated under conditions of nutrient limitation and elevated CO_2_ level (2500±100 ppm). Control, cultivated in 1/5 Hoagland medium. Pn, net photosynthetic rate. Error bars represent the SDs measured from three independent cultures. Asterisk indicates statistically significant difference between treatment group and control in the same assay conditions (Student’s *t*-test). *, P<0.05; **, P<0.01; ***, P<0.001.


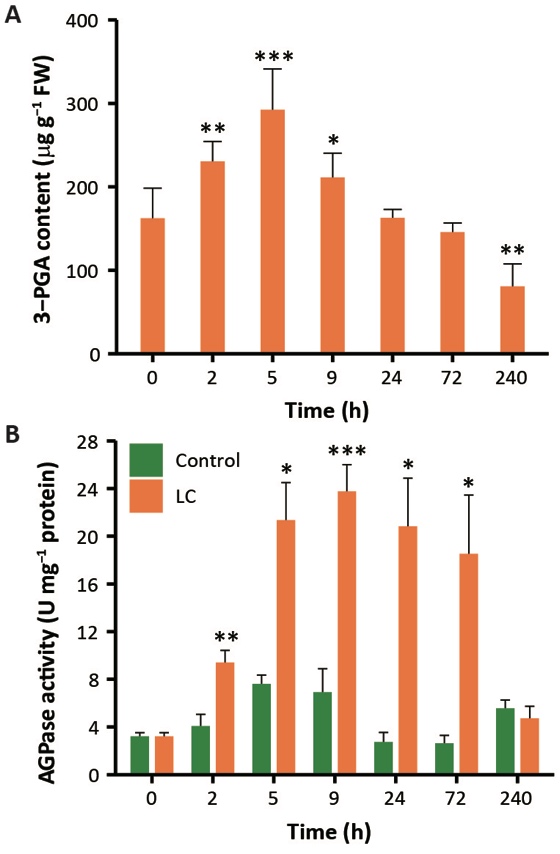


Figure S10.

Changes in 3-PGA content (**A**) and AGPase activity (**B**) in *Landoltia punctata* under LC treatment.

LC*,* cultivated under conditions of nutrient limitation and elevated CO_2_ level (2500±100 ppm). Control, cultivated in 1/5 Hoagland medium. 3-PGA, 3-phosphoglycerate. Error bars represent the SDs measured from three independent cultures. Asterisk indicates statistically significant difference between treatment group and 0 h (Student’s *t*-test). *, P<0.05; **, P<0.01; ***, P<0.001.


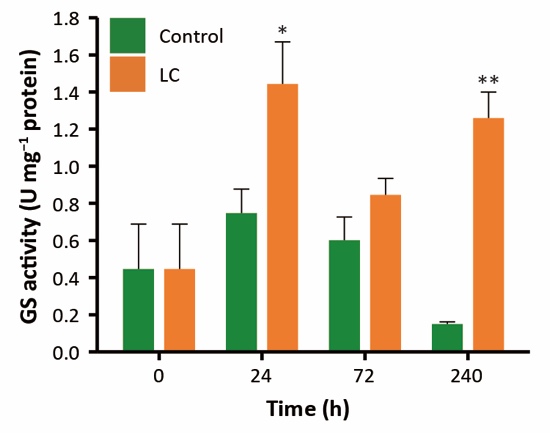


Figure S11.

Activity of GS in *Landoltia punctata*.

LC, cultivated under conditions of nutrient limitation and elevated CO_2_ level (2500 ± 100 ppm). Control, cultivated in 1/5 Hoagland medium. GS, glutamine synthase. Error bars represent the standard deviation measured from three independent cultures. Asterisks indicate statistically significant difference comparing data from each treatment group with control in the same assay conditions (Student’s *t*-test). *, P<0.05; **, P<0.01.


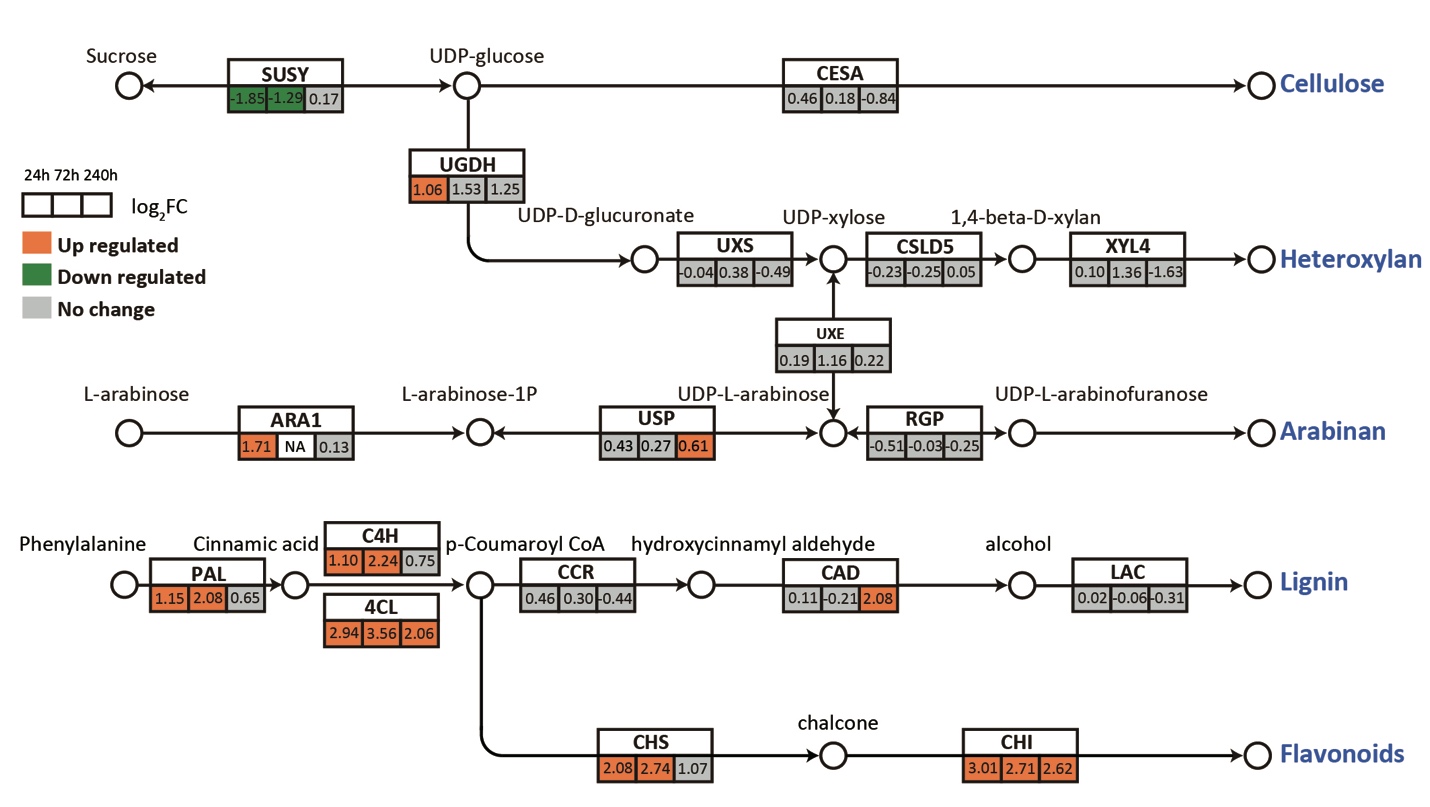


Figure S12.

Expression of key genes involved in biosynthesis of cellulose, hemicellulose, lignin, and flavonoids.

Numbers in boxs are log_2_(fold change) values at 24 h, 72 h and 240 h after nutrient limitation and elevated CO_2_ level compared with those at 0 h. Orange or green boxes indicate up- or down-regulated DEGs, respectively. Details are provided in Data S7 and Data S9.


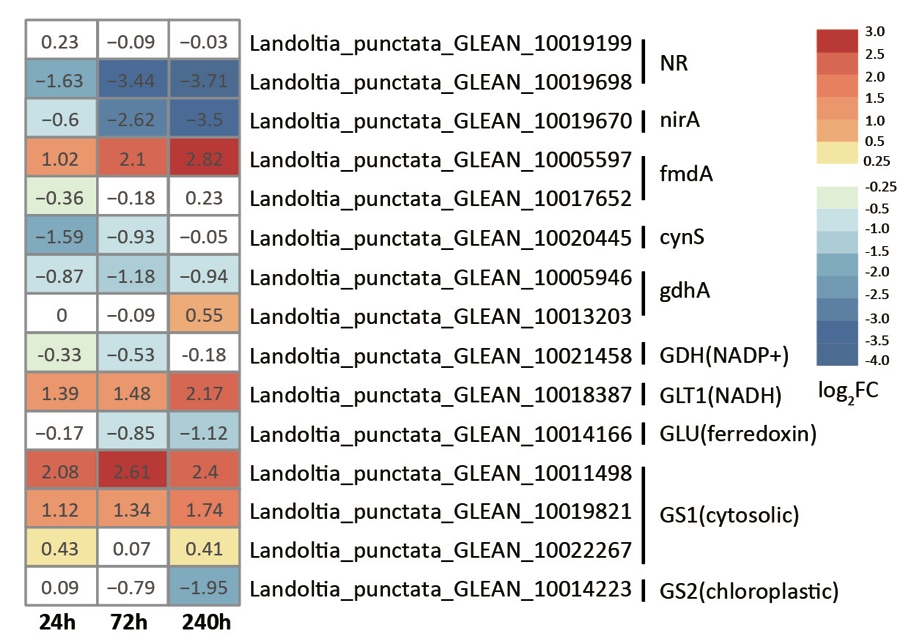


Figure S13.

Expression of genes involved in nitrogen assimilation of *Landoltia punctata*.

Numbers in the boxes are log_2_FC values at 24 h, 72 h and 240 h after nutrient limitation and elevated CO_2_ level compared with expression values (FPKM) with those at 0 h. Details provided in Data S12a.


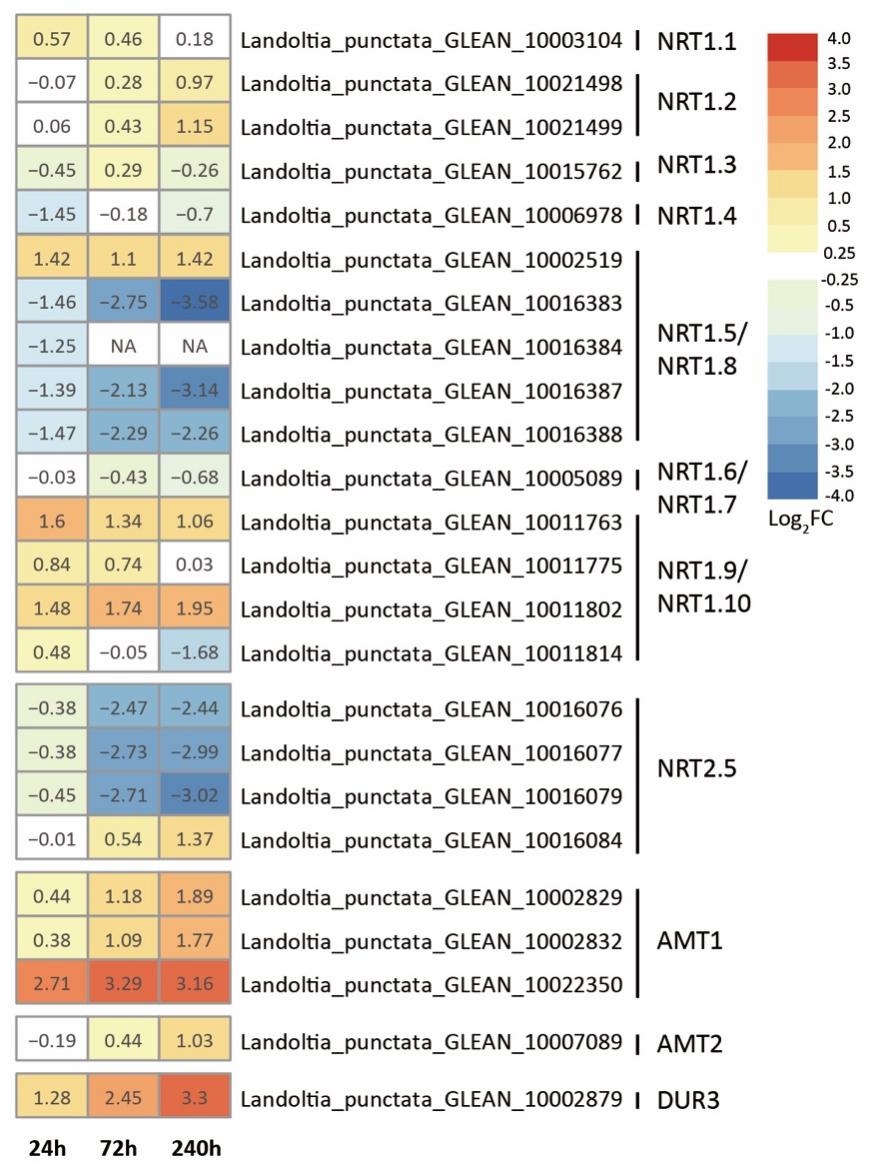


Figure S14.

Expression of genes involved in nitrogen transport of *Landoltia punctata*.

Numbers in the boxes are log_2_FC values at 24 h, 72 h and 240 h after nutrient limitation and elevated CO_2_ level compared with expression values (FPKM) with those at 0 h. Details provided in Data S12b.


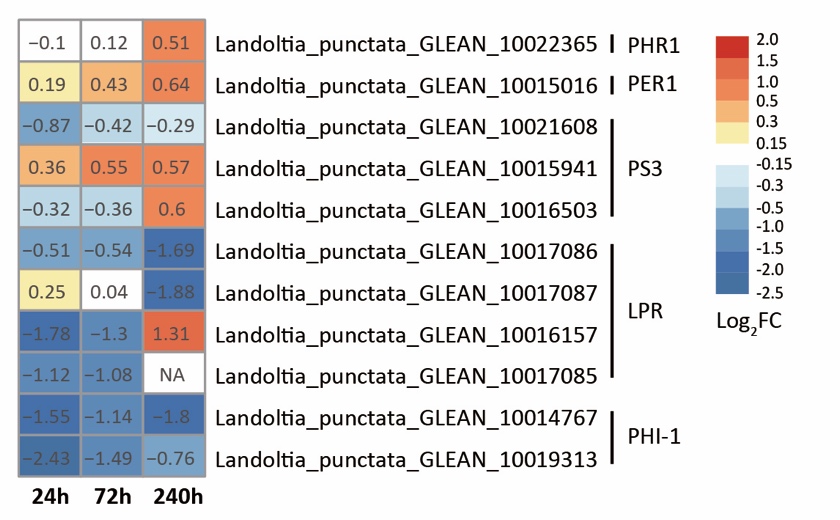


Figure S15.

Expression of genes involved in low phosphate response of *Landoltia punctata*.

Numbers in the boxes are log_2_FC values at 24 h, 72 h and 240 h after nutrient limitation and elevated CO_2_ level compared with expression values (FPKM) with those at 0 h. Details provided in Data S14a.


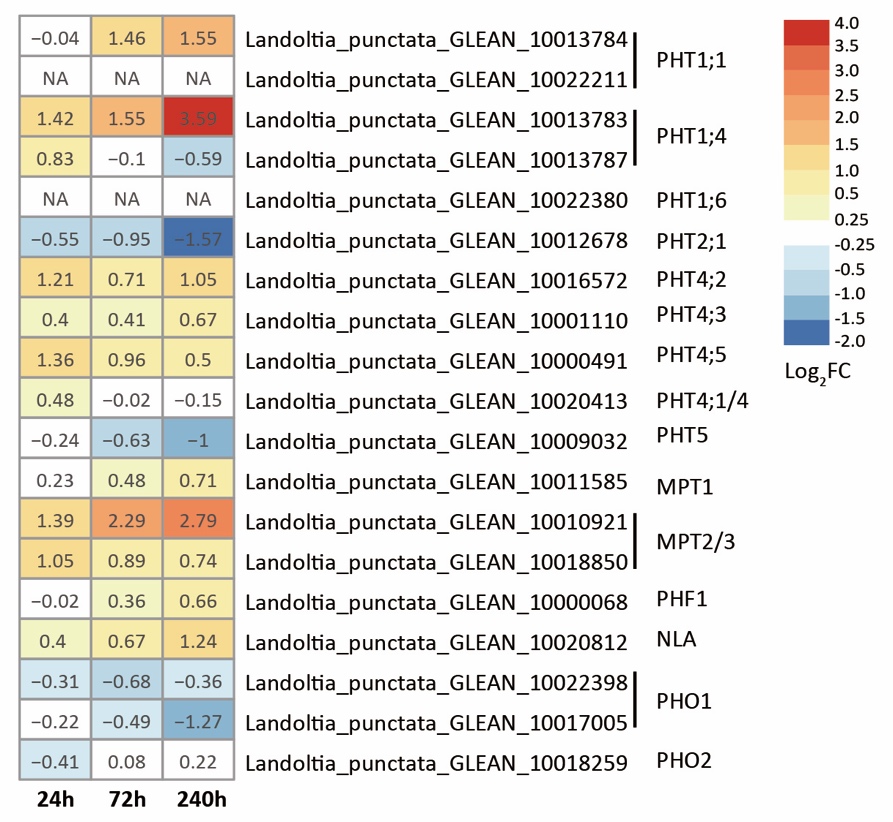


Figure S16.

Expression of genes involved in phosphate transport of *Landoltia punctata*.

Numbers in the boxes are log_2_FC values at 24 h, 72 h and 240 h after nutrient limitation and elevated CO_2_ level compared with expression values (FPKM) with those at 0 h. Details s provided in Data S14b.


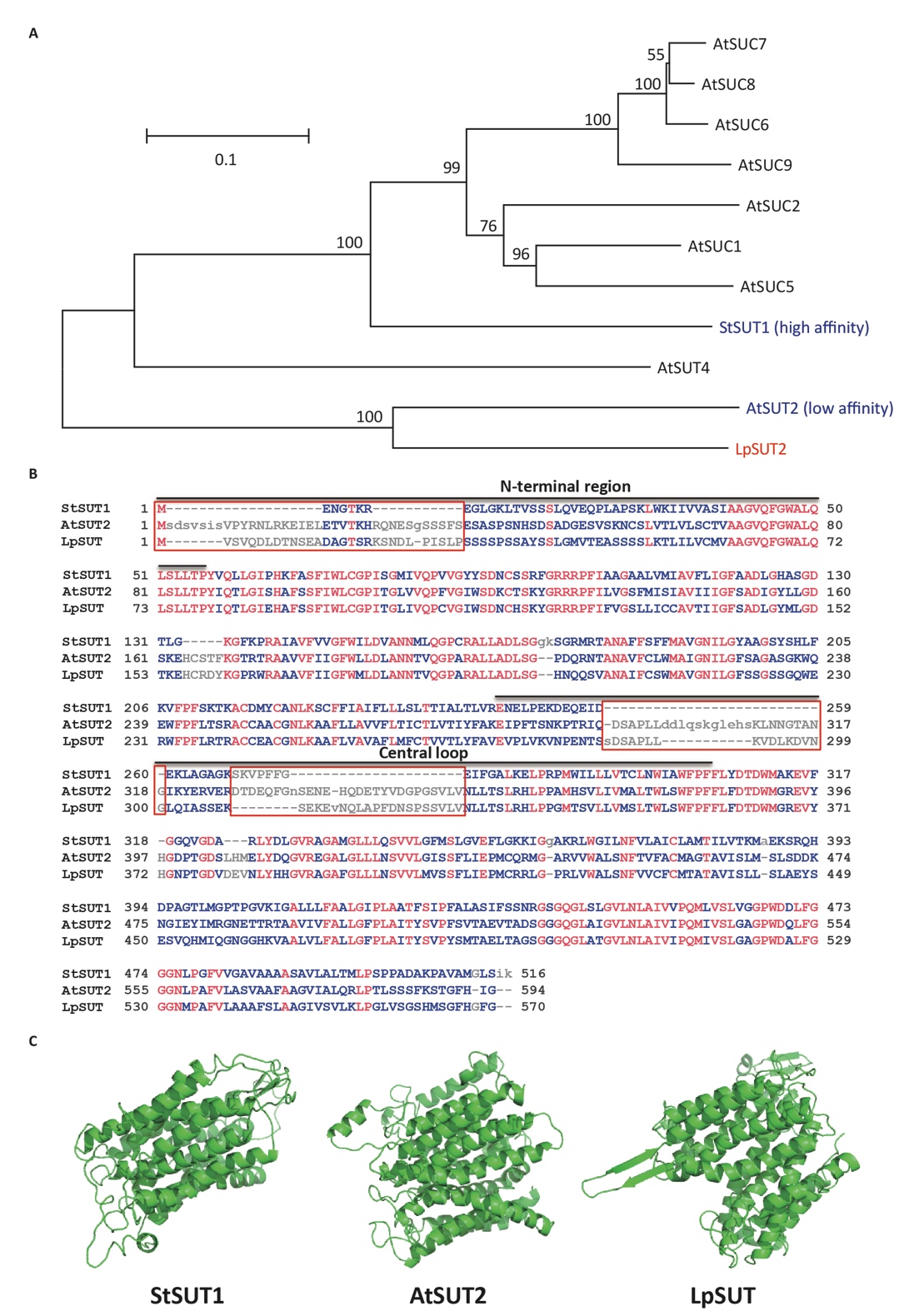


Figure S17.

SUT phylogenetic tree and sequence alignment among *Landoltia punctata* (Lp), *Arabidopsis thaliana* (At), and *Solanum tuberosum* (St).

**A**, SUT phylogenetic tree. Bar, substitution/site, 0.1.

**B**, Sequence alignment of SUT proteins among *Landoltia punctata* (*LpSUT*), *Arabidopsis thaliana* (*AtSUT2*, low-affinity sucrose transporter), and *Solanum tuberosum* (*StSUT1*, high-affinity sucrose transporter). Cytoplasmic extended domain at N-terminal region (first black line region) and extended domain at central loop region (second black line region) are marked in red boxes.

**C**, Predicted 3D structure of StSUT1, AtSUT2, and LpSUT.


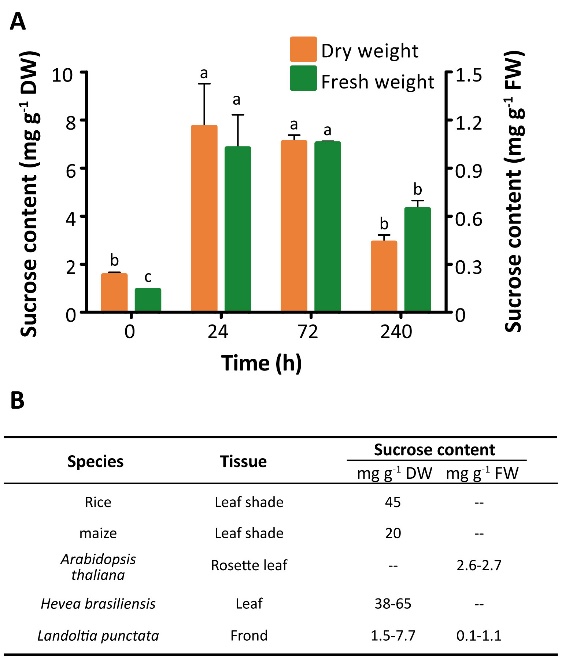


Figure S18.

Sucrose contents in *Landoltia punctata* under LC treatment.

**A**, Sucrose content in *Landoltia punctata*. Letters indicate significant differences among time points. Dry weights and Fresh weights are tested by one way ANOVA following Tukey-Kramer test (p<0.05).

**B**, Comparison of sucrose content in *Landoltia punctata* (determined in this study), rice (Xu et al., 2017), maize (Coneva et al., 2012), *Arabidopsis thaliana (Lukaszuk et al., 2017),* and *Hevea brasiliensis (Zhu et al., 2018)*.


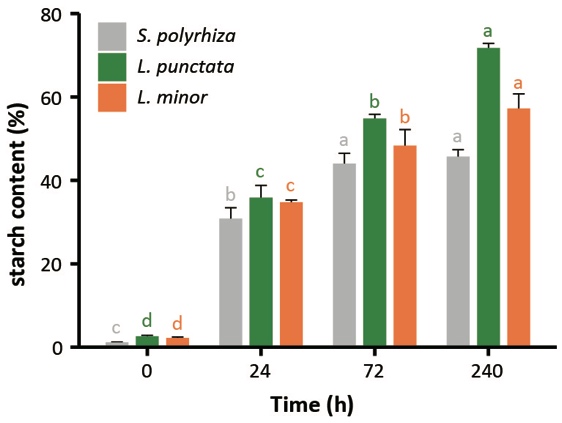


Figure S19.

Accumulation of starch in *Spirodela polyrhiza*, *Landoltia punctata*, and *Lemna minor* under LC treatment.

Error bars show standard deviations measured from three independent cultures. Letters indicate significant differences determined by one way ANOVA following Tukey-Kramer test (p<0.05).


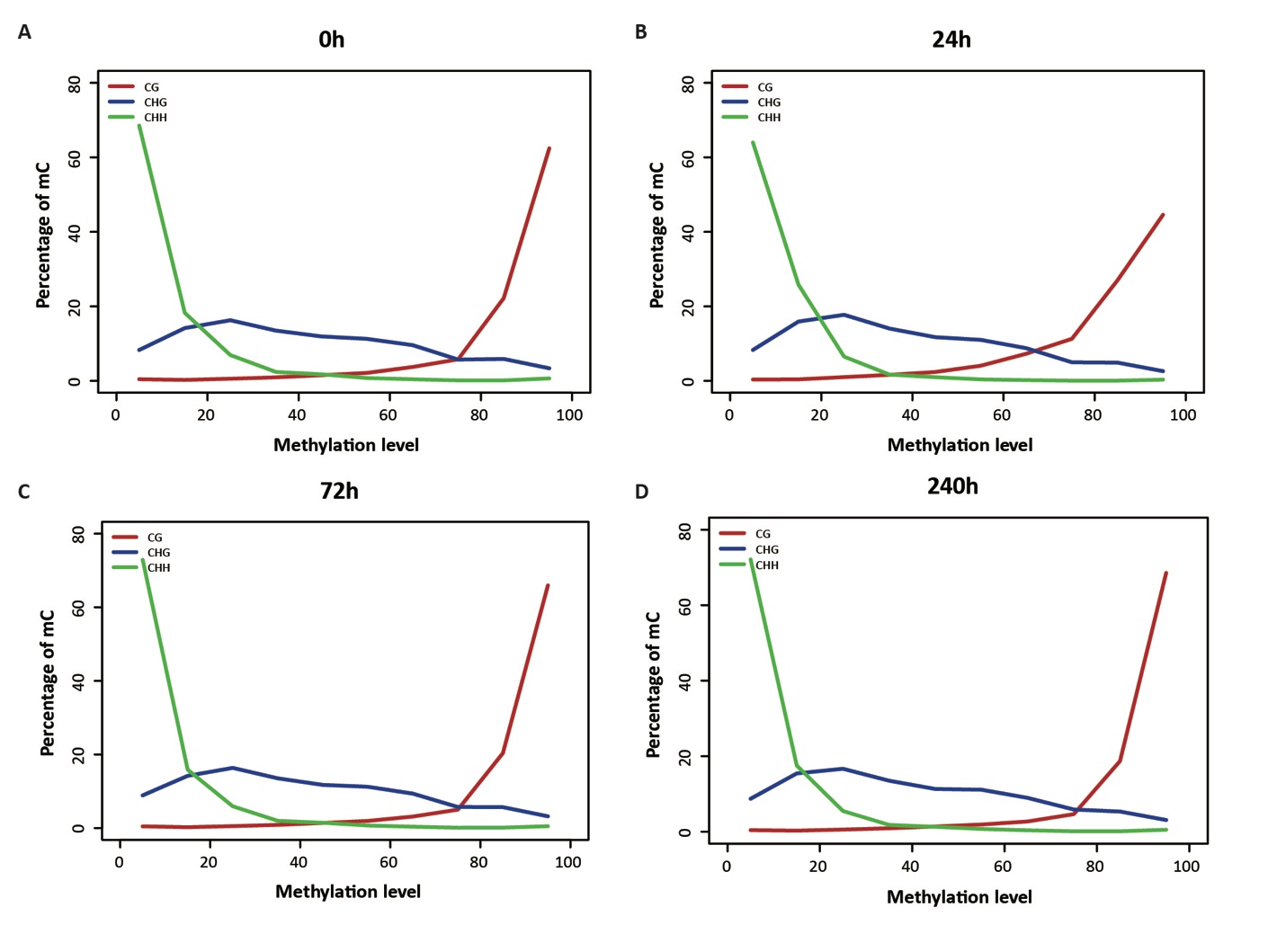


Figure S20.

Methylation level distribution of methylated cytosine (mC).

**
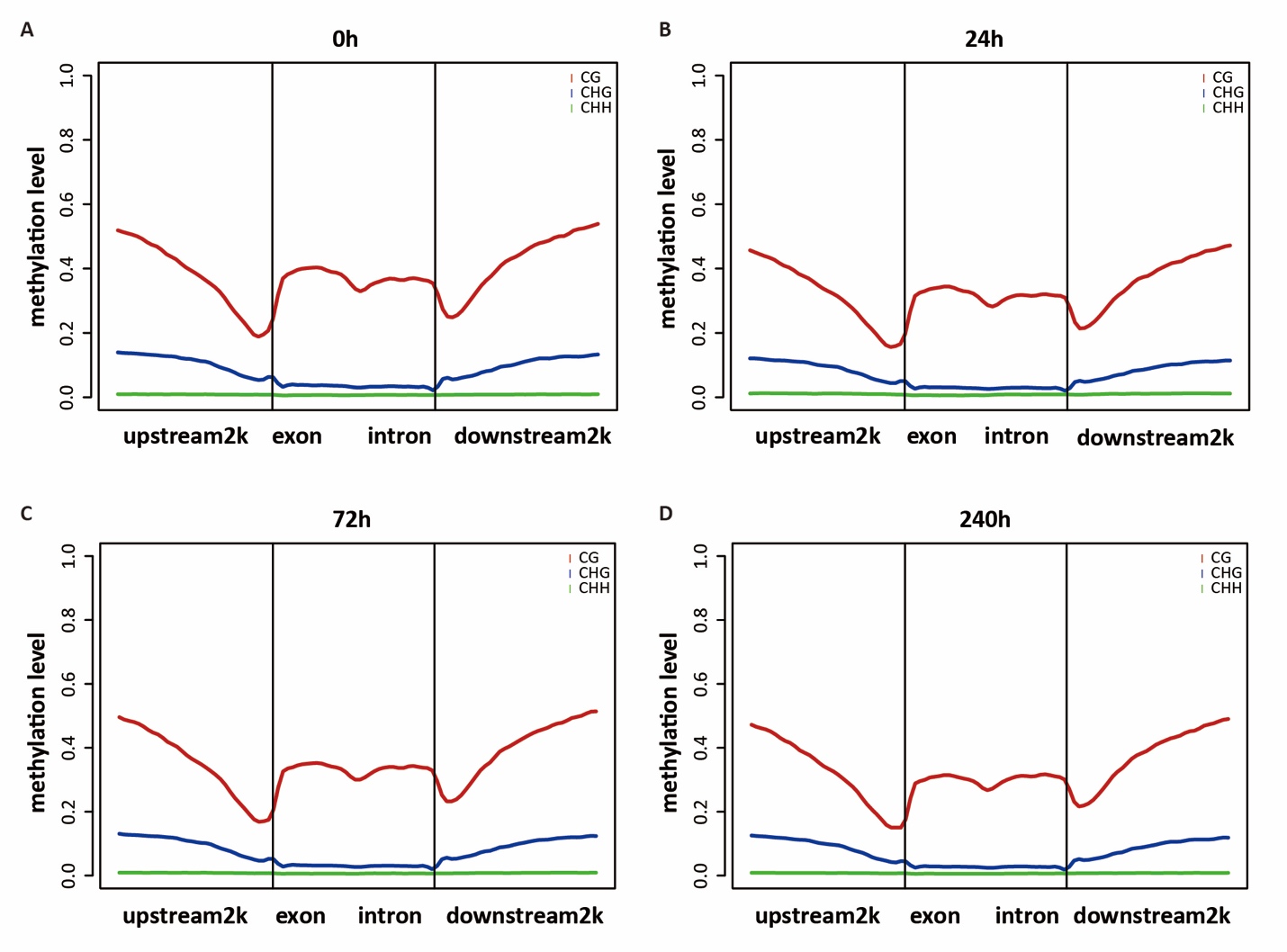
**

Figure S21.

The methylation level of different regions of genome. Upstream2k/downstream2k, the 2 kb-upstream or 2 kb-downstream regions of genes.

**
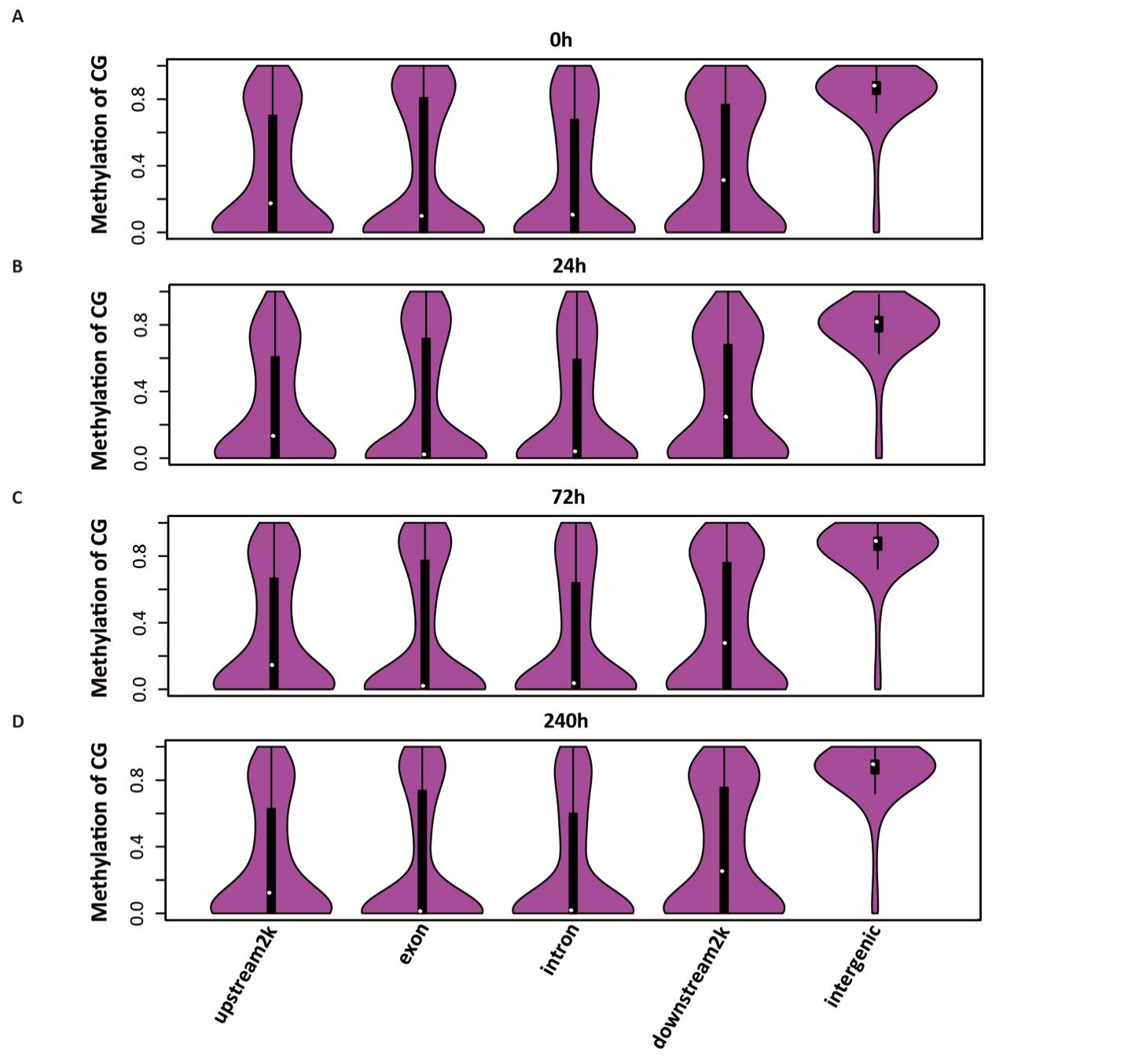
**

Figure S22.

Methylation level of CG in different regions of genome. Upstream2k/downstream2k, the 2 kb-upstream or 2 kb-downstream regions of genes.

Table S1.

Treatments for starch production by *Landoltia punctata*.

| Treatment | CO_2_ concentration (ppm) | Media |
| --- | --- | --- |
| Control | 390±50^2^ | 1/5 Hoagland |
| C^1^ | 2500±100 | 1/5 Hoagland |
| L^1^ | 390±50^2^ | Deionized water |
| LC^1^ | 2500±100 | Deionized water |

^1^ C: elevated CO_2_ level; L, nutrient limitation; LC, nutrient limitation and elevated CO_2_ level.

^2^ The CO_2_ concentration in atmosphere is 390±50 ppm.

Table S2.

Starch production ability of *Landoltia punctata* in pilot scale.

Fresh duckweed of approximately 4.0 kg was inoculated into 3.1×4.5×0.4 m^3^ (W×L×D) tanks filled with tap water. Duckweed was cultivated at 25ºC with sunlight in daytime and fluorescent lamp at night. CO_2_ was aerated to a concentration of 2500±100 ppm.

| Batch | 1 | 2 | 3 | 4 | Mean |
| --- | --- | --- | --- | --- | --- |
| Cultivation time (d) | 4 | 4 | 4 | 4 | - |
| Starch content (% DW) | 41.25 | 49.34 | 45.48 | 47.66 | 45.93±3.50 |
| Growth rate (g m^-2^ d^-1^) | 14.31 | 14.63 | 16.98 | 17.92 | 15.96±1.77 |
| Starch productivity (g m^-2^ d^-1^) | 8.55 | 9.24 | 10.89 | 11.48 | 10.04±1.37 |
| Moisture (%) | 84.35 | 81.12 | 83.47 | 83.14 | 83.02±1.36 |

Table S3.

RNA-Sequencing data.

| Sample ID | Raw bases  (Gb) | Clean bases  (Gb) | Read length  (bp) | Base Q20  (%) | GC content  (%) |
| --- | --- | --- | --- | --- | --- |
| LC0h_1 | 7.2 | 6.9 | 150×2 | 97.8 | 56.4 |
| LC0h_2 | 7.2 | 6.9 | 150×2 | 97.8 | 56.5 |
| LC0h_3 | 7.2 | 6.9 | 150×2 | 97.8 | 55.3 |
| LC24h_1 | 7.2 | 7.0 | 150×2 | 98.0 | 57.1 |
| LC24h_2 | 7.2 | 7.0 | 150×2 | 97.6 | 56.7 |
| LC24h_3 | 7.2 | 7.0 | 150×2 | 97.6 | 56.7 |
| LC72h_1 | 7.2 | 7.0 | 150×2 | 98.0 | 56.9 |
| LC72h_2 | 7.2 | 7.0 | 150×2 | 97.2 | 56.7 |
| LC72h_3 | 7.2 | 6.9 | 150×2 | 97.7 | 56.5 |
| LC240h_1 | 7.0 | 6.9 | 150×2 | 98.0 | 56.1 |
| LC240h_2 | 7.2 | 7.0 | 150×2 | 98.0 | 56.2 |
| LC240h_3 | 7.2 | 6.9 | 150×2 | 98.0 | 55.8 |

Table S4.

Primers for the selected DEGs for qRT-PCR.

F: Forward primer; R: Reverse primer.

| Gene ID | Primers (5'→3') | Products length (bp) |
| --- | --- | --- |
| Landoltia_punctata_GLEAN_10003767 | F: GGATCGGGCTGGTCGTTAC  R: GAATTCCCCCCTTCTTGCTG | 96 |
| Landoltia_punctata_GLEAN_10001924 | F: CATTGGTGCTGTTTTTTGAAGGG  R: TTCATCATTTGCAGCTACGATCC | 97 |
| Landoltia_punctata_GLEAN_10003150 | F: GAACTCCCACTCGCTGTTGTCT  R: CCTCTCGGTACTCTTCTCCCCT | 116 |
| Landoltia_punctata_GLEAN_10015586 | F: CGCCGTCCTTCAAACCAA  R: CCAATACGCCGGGCCATA | 148 |
| Landoltia_punctata_GLEAN_10000800 | F: TTTTTCAGGAGAGAGGTGTCCA  R: GGTTTGAGCAGTTTGATGGC | 128 |
| Landoltia_punctata_GLEAN_10004280 | F: TTCGCGACTCCTGTTTCGTT  R: ATACCTGGCGTGCGTGTTCA | 106 |
| Landoltia_punctata_GLEAN_10011855 | F: GCTATCTGCCCAAGGTCTGC  R: GGGGGTTCTCCACTTCCACT | 126 |
| Landoltia_punctata_GLEAN_10019540 | F: CAGCCGTTCACGTCTTTGGT  R: TGCCGTCCTCGTCCTTCTTA | 114 |
| Landoltia_punctata_GLEAN_10019599 | F: AGCAAGAGCCCCGTCACC  R: CCGGCGCAACTTCCAAT | 113 |
| Landoltia_punctata_GLEAN_10000438 | F: CCTCGTTTTGGGTTGTAATATTC  R: GGCAAGGGACCTCCTTTGGC | 104 |
| Landoltia_punctata_GLEAN_10012804 | F: ATGCTTTGACAGAGCGTGTTG  R: GTTGCTTAGGCTGCCAGTG | 121 |
| Landoltia_punctata_GLEAN_10000828 | F: TTTCGGGCTGTTCGGGTTG  R: GGCGATGATGGCGTTCTTCT | 94 |
| Landoltia_punctata_GLEAN_10018179 | F: TGCCGTCCTCGCCTTACTG  R: CCGCAGCCGTTCACATCTT | 117 |
| Landoltia_punctata_GLEAN_10004572 | F: GGGAAAAAAGTCGAGCATACC  R: TCGTTGCCCACAGTGAAAATA | 133 |
| Landoltia_punctata_GLEAN_10010520 | F: TAGCGGAGTAAAATCGGTGACG  R: GGGGAAATGAAAAATGGAGAAA | 116 |
| Landoltia_punctata_GLEAN_10019316 | F: ACCCAACTCCCTGTCATTTTTCT  R: CCTGCTTTATCCGGTTCCATC | 109 |
| Landoltia_punctata_GLEAN_10011880 | F: TCTCTCGTGCTTTCTTCGCTC  R: GCACACCTTCTTCCACTCCATC | 90 |
| Landoltia_punctata_GLEAN_10012751 | F: GTCGCTGTGATGCTGGTTTTC  R: TTCCCGTGTACATGACGTTGC | 133 |
| Landoltia_punctata_GLEAN_10014601 | F: CTCGGCGAGGAACACGCT  R: TCCCAGATGCCCAAACGG | 140 |
| Landoltia_punctata_GLEAN_10018050 | F: TACGGGGAGGGAGAACCC  R: CGCCGACGACGACAACA | 133 |
| *Actin* (Internal control) | F: TGATGGTTGGAATGGGACAG  R: TTGGTCACAACGCCATGCT | 106 |

Table S5.

Primers for quantification of the key genes’ expression involved in CO_2_ fixation, carbon concentration, and starch synthesis by qRT-PCR.

F: Forward primer; R: Reverse primer.

| Gene name | Primers (5'→3') | Products length (bp) |
| --- | --- | --- |
| *Actin* (Internal control) | F: TGATGGTTGGAATGGGACAG  R: TTGGTCACAACGCCATGCT | 106 |
| *AGPase* | F: ATCACGCATCCTTCACCAATC  R: TCATCTCCAATCTACACTCAACCT | 95 |
| *SSS* | F: TATGGCACGGAAGAGTTGAAG  R: TCTCTCTGGCGAAGGAACTCA | 151 |
| *Rubisco* | F: GGTGGAGGAGGTCAAGAAGG  R: GCTTGGCTGCAATGAAACTG | 101 |
| *PEPC* | F: GAGATGAGAGCGGGGATGAG  R: TGAGAGGAGCGTTGTAGGGG | 130 |
| *GBSS* | F: GGACCCCAACGCTCTTCTCTT  R: CGACCATCTGGATGTTCTCGC | 113 |
| *UGPase* | F: TTGAAGGTATCTGGGGATGTGTG  R: GAGTTGACTTGTTCTCGAGGGTG | 127 |

Table S6.

WGBS data of *Landoltia punctata* under LC treatment.

Samples were withdrawn at time points of 0 h, 24 h, 72 h, and 240 h, which were marked as LC_0 h, LC_24 h, LC_72 h, and LC_240 h, respectively. RL, read length; *R*_a_, align rate; *R*_nd_, nonDup rate; *R*_c_, conversion rate.

| Samples | Raw reads | Clean reads | RL  (bp) | Q20  (%) | Align reads | *R*_a_  (%) | nonDup reads | *R*_nd_  (%) | *R*_c_  (%) |
| --- | --- | --- | --- | --- | --- | --- | --- | --- | --- |
| LC_0 h | 165,497,180 | 151,982,152 | 150×2 | 99.7 | 131,841,668 | 86.8 | 119,789,431 | 90.9 | 96.9 |
| LC_24 h | 178,456,582 | 165,815,374 | 150×2 | 99.6 | 128,862,610 | 77.7 | 120,079,561 | 93.2 | 96.7 |
| LC_72 h | 195,165,098 | 179,820,248 | 150×2 | 99.7 | 158,551,840 | 88.2 | 143,195,052 | 90.3 | 94.5 |
| LC_240 h | 204,367,354 | 189,216,976 | 150×2 | 99.7 | 166,590,668 | 88.0 | 150,834,753 | 90.5 | 95.5 |

Table S7.

The global methylation level of *Landoltia punctata* under LC treatment.

Samples were withdrawn at time points of 0 h, 24 h, 72 h, and 240 h, which were marked as LC_0 h, LC_24 h, LC_72 h, and LC_240 h, respectively.

| Samples | C (%) | CG (%) | CHG (%) | CHH (%) |
| --- | --- | --- | --- | --- |
| LC_0 h | 12.9 | 76.7 | 22.0 | 1.4 |
| LC_24 h | 11.2 | 70.6 | 19.7 | 1.8 |
| LC_72 h | 13.0 | 77.4 | 21.9 | 1.3 |
| LC_240 h | 12.9 | 78.1 | 22.2 | 1.3 |

Table S8.

Links for genomes used in this research.

| Species | Links |
| --- | --- |
| *Klebsormidium flaccidum* | http://www.plantmorphogenesis.bio.titech.ac.jp/~algae_genome_project/klebsormidium/kf_download.htm |
| *Spirodela polyrhiza* | ftp://ftp.ncbi.nlm.nih.gov/genomes/all/GCA/001/981/405/GCA_001981405.1_ASM198140v1/GCA_001981405.1_ASM198140v1_genomic.gff.gz |
| *Landoltia punctata* | https://www.ncbi.nlm.nih.gov/bioproject/PRJNA546087 |
| *Zostera marina* | ftp://ftp.ncbi.nlm.nih.gov/genomes/all/GCA/001/185/155/GCA_001185155.1_Zosma_marina.v.2.1/GCA_001185155.1_Zosma_marina.v.2.1_genomic.gff.gz |
| *Lemna minor* | https://genomevolution.org/GenomeInfo.pl?gid=27419 |
| *Arabidopsis thaliana* | ftp://ftp.ncbi.nlm.nih.gov/genomes/all/GCF/000/001/735/GCF_000001735.3_TAIR10/GCF_000001735.3_TAIR10_genomic.gff.gz |
| *Oryza sativa* | ftp://ftp.ncbi.nlm.nih.gov/genomes/all/GCF/000/005/425/GCF_000005425.2_Build_4.0/GCF_000005425.2_Build_4.0_genomic.gff.gz |
| *Zea mays ssp. mays* | ftp://ftp.ncbi.nlm.nih.gov/genomes/all/GCF/000/005/005/GCF_000005005.2_B73_RefGen_v4/GCF_000005005.2_B73_RefGen_v4_genomic.gff.gz |

**References**

Coneva, V., Guevara, D., Rothstein, S. J., and Colasanti, J. (2012). Transcript and metabolite signature of maize source leaves suggests a link between transitory starch to sucrose balance and the autonomous floral transition. *Journal of Experimental Botany* 63, 5079-5092. doi: 10.1093/jxb/ers158

Krugel, U., and Kuhn, C. (2013). Post-translational regulation of sucrose transporters by direct protein-protein interactions. *Frontiers in Plant Science* 4, 237. doi: 10.3389/fpls.2013.00237

Lukaszuk, E., Rys, M., Mozdzen, K., Stawoska, I., Skoczowski, A., and Ciereszko, I. (2017). Photosynthesis and sucrose metabolism in leaves of Arabidopsis thaliana aos, ein4 and rcd1 mutants as affected by wounding. *Acta Physiologiae Plantarum* 39. doi: 10.1007/s11738-016-2309-1

Smith, A. M., Zeeman, S. C., and Smith, S. M. (2005). Starch degradation. *Annual Review of Plant Biology* 56, 73-98. doi: 10.1146/annurev.arplant.56.032604.144257

Xu, Y. H., Sechet, J., Wu, Y. B., Fu, Y. P., Zhu, L. F., Li, J. C., et al. (2017). Rice Sucrose Partitioning Mediated by a Putative Pectin Methyltransferase and Homogalacturonan Methylesterification. *Plant Physiology* 174, 1595-1608. doi: 10.1104/pp.16.01555

Zhu, J. H., Qi, J. Y., Fang, Y. J., Xiao, X. H., Li, J. H., Lan, J. X., et al. (2018). Characterization of Sugar Contents and Sucrose Metabolizing Enzymes in Developing Leaves of Hevea brasiliensis. *Frontiers in Plant Science* 9. doi: 10.3389/fpls.2018.00058
